# Characteristics of Kundalini-Related Sensory, Motor, and Affective Experiences During Tantric Yoga Meditation

**DOI:** 10.3389/fpsyg.2022.863091

**Published:** 2022-06-30

**Authors:** Richard W. Maxwell, Sucharit Katyal

**Affiliations:** ^1^Private Practitioner, Ithaca, NY, United States; ^2^Independent Researcher, Copenhagen, Denmark

**Keywords:** meditation, tantric yoga, kundalini, subjective experiences, anomalous experiences, Ananda Marga, contemplative practices

## Abstract

Traditional spiritual literature contains rich anecdotal reports of spontaneously arising experiences occurring during meditation practice, but formal investigation of such experiences is limited. Previous work has sometimes related spontaneous experiences to the Indian traditional contemplative concept of kundalini. Historically, descriptions of kundalini come out of Tantric schools of Yoga, where it has been described as a “rising energy” moving within the spinal column up to the brain. Spontaneous meditation experiences have previously been studied within Buddhist and Christian practices and within eclectic groups of contemplative practitioners. Prior explorations of kundalini have emphasized extreme experiences, sometimes having clinical consequences. We conducted a first such investigation of kundalini-related experiences within a sample of meditators from a single Tantric Yoga tradition (known as Ananda Marga) that emphasizes the role of kundalini. We developed a semi-structured questionnaire to conduct an exploratory pilot investigation of spontaneous sensory, motor and affective experiences during meditation practice. In addition to identifying the characteristics of subjective experiences, we measured quantity of meditation, supplemental practices, trait affect and trait mindfulness. We administered it to 80 volunteers at two Ananda Marga retreats. Among reported experiences, we found the highest prevalence for positive mood shifts, followed by motor and then sensory experiences. The frequency of spontaneous experiences was not related to the quantity of practiced meditation or trait measures of mindfulness and affect. Self-reports included multiple descriptions of rising sensations, sometimes being directly called kundalini. Experiences with rising sensations were complex and many included references to positive affect, including ecstatic qualities. There were also reports of spontaneous anomalous experiences. These experiences of rising sensations resemble prior clinical descriptions that were considered kundalini-related. The individuals who reported rising sensations could not be distinguished from other participants based on the incidence of experiences, quantity of meditation practice, or trait measures of mindfulness and affect. In contrast, greater amount of Tantric Yoga meditation practice was associated with greater positive affect, less negative affect and greater mindfulness. Further study of these exploratory findings and how they may be related to spiritual and well-being goals of meditation is warranted along with scientific investigation of purported kundalini phenomena.

## Introduction

In recent years, there has been growing interest in the lived experience of meditation (Lutz et al., 2015; Shaner et al., 2017; Kordeš et al., 2019; Petitmengin et al., 2019; Agarwal et al., 2020). Subjective experiences during meditation have been examined along several dimensions including, the quality or depth of meditation (Piron, 2001; van Lutterveld et al., 2017; Katyal and Goldin, 2021), and changes in experiential boundaries of self (Lindahl and Britton, 2019; Berkovich-Ohana et al., 2020; Nave et al., 2021). Recent work has also revealed that meditation practice may engender experiences that have strong personal significance and change personal outlooks, often referred to as “kundalini awakening” (Lockley, 2019; Corneille and Luke, 2021; Woollacott et al., 2021). Tantric Yoga practices, within which the concept of kundalini originated (Hatley, 2022), have long been known to generate such experiences in the process of deepening meditation (Silburn, 1988; White, 2012).

Kundalini is a complex concept, first appearing in *Tantric Śaivism* scriptures *circa* 6–8th century C.E. It refers to a dormant “vital power” that rises up the spinal column when “aroused,” sometimes yielding strong transformative personal experiences (Hatley, 2022). Despite some variations in conceptualizations of kundalini across traditional scriptures, a shared feature is the idea that it ascends through a central energy channel, traditionally referred to as the *sushumna nadi*, situated within the spine (Mallinson and Singleton, 2017). Along this channel, Tantric Yoga literature has described subtle energy centers (referred to as “chakras”), which are bodily experienced locations through which the kundalini is proposed to ascend (Anandamurti, 1993b). The most commonly-known chakra system includes seven chakras (Feuerstein, 1997), though there are slight differences of opinion concerning the precise bodily locations where these chakras are supposed to lie (e.g., Motoyama, 1981; Vishnu Tirtha, 2000; Saraswati, 2006; Anandamurti, 2010b). Our participants primarily utilize the classic seven chakra system described by Anandamurti (2010b), which is supplemented by two additional chakras (details in section “Materials and Methods”).

The ascent of the kundalini has been associated with experiential phenomena in sensory modalities, including light, heat, somatosensory and different kinds of inner sounds (Feuerstein, 1997; Anandamurti, 2020a,c), motor activity (Vishnu Tirtha, 2000), and affect (Anandamurti, 1968). While kundalini has traditionally been considered a non-physical “force” capable of being aroused (Anandamurti, 1993a), kundalini has never been directly measured. As a result, kundalini’s association with experiential phenomena is presumed to be indirect. Therefore, we use the term “kundalini-related” phenomena for experiences that may be measurable or reportable and which have shown some relationship with identified patterns of experiences associated with kundalini.

A framework for formal investigation of kundalini-related phenomena was developed in a psychiatric context (Sannella, 1992). Sannella organized the “signs” (objective indications) and “symptoms” (subjective descriptions) of what he called “physio-kundalini” experiences into four basic categories: motor, sensory, interpretive (i.e., affective and cognitive), and non-physiological (parapsychological or anomalous) phenomena, while recognizing that these phenomena might be “different aspects of a single integrated experience” (1992, p. 93). Motor phenomena included a range of spontaneous movements from simple twitching, to prolonged trembling and automatic assumption of complex yogic postures; audible vocal outbursts; unusual breathing patterns, including rapid breathing, shallow breathing, deep breathing, and extended breath retention; and frozen postures resembling paralysis. Sensory phenomena included somatosensory (tingling, tickling, itching, and vibrating sensations); temperature (heat and cold sensations); inner lights and visions; and inner sounds of many varieties. Interpretive phenomena represented unusual or extreme emotion; distortions of thought process; detachment from typical thoughts, feelings and sensations; dissociation from one’s own mental processes, potentially leading to delusions; what was called “single seeing” (a subjective state with a unitary perceptive origin); and a “great body” experience in which the kinesthetic sense seems to extend beyond the normal boundaries. Non-physiological phenomena included out of body experiences and paranormal “psychic” perceptions.

More recently, to measure kundalini-related phenomena, researchers have developed a Kundalini Awakening Scale (KAS; Sanches and Daniels, 2008). The KAS consists of 76 items organized into five subscales (Changes, Negative experiences, Positive experiences, Involuntary positionings, and Physical symptoms) and a Total score. The KAS items were generated from four primary sources: an extensive personal description of presumed kundalini effects (Krishna, 1993), clinical data (Sannella, 1992), a theoretical conception (Bentov, 1988), and spiritual emergency work (Grof and Grof, 1992), along with some additional unspecified reports of kundalini awakening.

A few studies have examined the effects of meditation on KAS. Individuals engaging in a relatively unstructured set of meditation practices were found to have a positive association between negative experiences and the overall KAS scores (Sanches and Daniels, 2008). In a primarily student population, greater weekly time spent practicing meditation was associated with higher KAS Total scores (de Castro, 2015). The presence of greater KAS Physical symptoms scores (such as, unusual or unexpected tactile and temperature sensations, or unusual breathing and muscle activity) was considered a possible distinguishing feature of kundalini-related involvement in spiritually transformative experiences (Corneille and Luke, 2021). Additional scales associated with kundalini experiences have been developed in the context of clinical conditions (Greyson, 1993a; Thalbourne and Fox, 1999).

Besides the positive effects classically associated with meditation, recent studies have also investigated adverse meditation experiences (Cebolla et al., 2017; Anderson et al., 2019; Schlosser et al., 2019; Farias et al., 2020; Goldberg et al., 2021; Lambert et al., 2021). According to traditional Tantric Yoga accounts, adverse experiences during meditation are considered to arise primarily due to the premature or improper arousal of kundalini, i.e., in meditators lacking adequate preparation (Krishna, 1993; Feuerstein, 1998). In contrast, we were interested in examining kundalini-related experiences in a sample that would have had adequate preparation for kundalini experiences. Because the concept of kundalini has traditionally been closely related to Tantric schools of meditation, we focused on recruiting a sample that was adept in such a system of meditation practice (Anandamurti, 1968). The primary difference between the experience of an improper arousal and a balanced arousal of kundalini may be the presence of a pre-existing psychiatric vulnerability that is more readily expressed when the proper training and support are not present (Suchandra et al., 2021). Qualities of experiences may have similarities for both improper and balanced arousal conditions, but in a kundalini syndrome (Valanciute and Thampy, 2011; Benning et al., 2018), they will have more prominent disruptive and unpleasant features. We focused specifically on meditators from Ananda Marga, a school of Tantric Yoga (Corby et al., 1978), because meditators of this school receive precise meditation instructions that are largely similar across individuals. We expected that this would provide a clearer impression of kundalini-related expression over prior studies which have examined experiences arising from eclectic sources with no common practices or philosophy (Taylor and Egete-Szabo, 2017; Lockley, 2019; Corneille and Luke, 2021; Woollacott et al., 2021). We predicted our sample would have a comparatively low incidence of adverse experiences resulting from the comprehensive practices and systematic training.

Because previous measures of kundalini-related experiences have been largely based on clinical reports involving people in distress (Greyson, 1993a; Thalbourne and Fox, 1999; Sanches and Daniels, 2008), they appeared somewhat inadequate for measuring such experiences in a healthy meditating sample of Tantric Yoga meditators. Therefore, rather than using pre-existing scales, we adopted the general experiential framework identified by Sannella (1992), and adapted it so that it could be used in a healthy sample versed with the idea of kundalini and engaging practices to awaken it. The majority of our questionnaire utilized a semi-structured format that allowed open-ended descriptions of participants’ meditation experiences in different sensory, motor and affective modalities. Additionally, we used standard questionnaires to measure traits of positive and negative affect, and trait mindfulness. We utilized reports of rising sensations involving the back as a primary marker for the presence of kundalini-related activity.

Furthermore, based on prior research (de Castro, 2015), we expected that participants with longer daily meditation practice would report greater kundalini-related experiences and greater mindfulness. We also predicted that greater reported years of meditation practice would correlate positively with trait positive affect and trait mindfulness scores, consistent with prior findings (Easterlin and Cardeña, 1998).

## Materials and Methods

### Participants

A total of 84 individuals were recruited and returned surveys. Recruitment occurred at two Ananda Marga meditation retreats. Two individuals were excluded due to incomplete surveys. Another survey lacked specification of the amount of daily meditation performed and was excluded. One additional individual was excluded because they were below 18 years of age. As a result, 80 participants (*n* = 33 females, 47 males) were included in this study. The mean age of the participants was 41.9 years (SD 13.9, range 20–77). Concerning education, 20 (25%) participants had a high school education, 6 (8%) had Associate’s degrees, 28 (35%) had Bachelor’s degrees, 16 (20%) had Master’s degrees, 7 (9%) had doctoral or law degrees, and 3 (4%) did not report their education.

All participants provided consent according to guidelines recommended by the American Psychological Association Ethics Code, section 8.02 (American Psychological Association, 2002). All participants volunteered and completed the surveys at their own pace during their personal time. Participants were allowed to skip items in the survey or opt-out of the study at any point, if they desired.

### Questionnaire Contents

After the consent form, the hand-written questionnaire began with demographic questions, followed by questions concerning the quantity of meditation performed and the degree of observance of various supplementary practices (see below). A series of questions examining experiences during meditation in any of six modalities was presented next. The six modality categories included four sensory realms (Somatosensory, Temperature, Light, and Sound), Motor activity and Mood. However, within the questionnaire, the term “Touch” was used instead of “Somatosensory” because we expected all individuals might not understand the meaning of “somatosensory.” With each sensory modality, participants were asked not to report any experiences that could be explained by an external sensory input. For the Motor modality, all reported experiences were asked to be movements that were spontaneous and unintentional. For Mood, participants were asked to describe any “unusual shift in mood” that was associated with meditation. At the end, two published scales were included to measure trait mindfulness and trait affect. Some additional items were present in the questionnaire that were not included in this analysis.

While kundalini was the central focus of this research, there was no use of the terms “kundalini” or “awakening” or “energy” in any information provided to the participants in order to avoid creating a bias or expectation. Information about self-transformations, parapsychological and anomalous experiences was not specifically sought, but we did analyze them when they were provided. We emphasized open-ended subjective responses so that descriptions could be as elaborate and varied as participants desired. Evaluating the content of subjective experience has been shown to provide more extensive information than just noting the presence of the category of the experience (Benning et al., 2018).

The questionnaire section called “Sensory and Muscular Experiences During Meditation,” contained the questions for reports of experiences in all of these six modalities. If any experience was present for a particular modality, participants were directed to additional questions which sought to clarify relevant characteristics. For example, for light, participants were initially asked, “Have you had any experiences with light, unrelated to external light sources, that you attribute to being a meditator?” If an answer of “yes” was provided, then they were asked, “Have you experienced light inside your head or body during meditation?” In addition to the “yes/no” components, participants were asked to describe what they experienced. Additional questions associated with frequency, strength, duration, location, color and form followed. Responses were grouped for some sets of descriptive responses based on inherent relationships, such as degrees of brightness, or quantity of time. The additional questions for each modality were different, designed to identify details that were unique to that modality.

For somatosensory responses, it was initially asked, “Have you had sensations such as pressure, tension, tickling, tingling, vibrating, quivering, itching, or crawling—pleasant or unpleasant—within the body or skin (independent of any normal cause)?” The designation of physical location was important for Somatosensory responses and part of the additional questions. Participants used a variety of different terminologies, particularly when using chakras to designate a location. Questions concerning chakras did not distinguish between the location of the controlling points and the concentration points. Sanskrit and English names for the same chakra were tallied together (e.g., root chakra and *muladhara* chakra were considered the same for analysis). Body regions not directly referring to a chakra were tallied separately. There were three related questions about somatosensory experiences. We combined these into a composite score, eliminating redundancies (if the same sensation was reported for more than one of the questions for a participant, it was only counted as one occurrence for that participant). It was also asked if the “physical sensations ever move through a sequence of locations (independent of you intention).” If so, they were asked to explain the progression.

For temperature, it was initially asked, “Independent of any defined medical condition or external source, have you ever seen a reddening of your skin, or sensed unusual heat or cold in your body, or on your skin?” For muscle activity, it was asked, “Have you ever experienced spontaneous involuntary movements, vocalizations, or breathing patterns independent of any obvious physical or psychological cause?” All modalities, except mood and sound, followed a questionnaire structure similar to the one explained for light. For mood, it was simply asked, “Have you ever experienced an unusual shift in mood that you associate with meditation, or other spiritual practices?” A request followed to explain whatever was experienced. For sound, it was asked, “Have you ever heard any sounds that appeared unrelated to any external source and that you attribute to being a meditator?” This was followed by asking if it occurred during meditation and then a designation the frequency of occurrence of specific types of sounds was requested. For those sounds, we utilized sound categories based on a sequence described in spiritual literature (Anandamurti, 1999). This literature suggests that, for some individuals, each cakra can be associated with a different sound as the kundalini rises from lower to higher chakras (from the *muladhara* to the *ajina* chakra; the sounds of crickets, ankle bells, sweet flute, gong or ocean, and om/aum, respectively). Sound options included items of that sequence, plus additional options (tones, music, hissing, roaring, and thunder) in order to introduce alternatives. Participants could also independently name two additional sounds.

To measure trait mindfulness, we used the total score of the CAMS-R: Cognitive and Affective Mindfulness Scale-Revised (Feldman et al., 2007). There was a significant correlation of gender with CAMS-R Total Score (*r* = 0.19, *p* = 0.007), but not age. Despite that, the CAMS-R had “good” internal consistency (alpha = 0.76) using definitions based on alpha levels adjusted for sample size and number of items (Pontekotto and Ruckdeschel, 2007). Multiple components of the CAMS-R had significant relationships with other measures of mindfulness, distress, well-being, emotional regulation, approaches to problems and other mindfulness measures, demonstrating convergent and discriminant validity. The CAMS-R is divided into four subscales (attention, present focus, awareness, and acceptance), which produce the total score when summed. The items were rated on a 4-point Likert scale from 1 (Rarely/Not at all) to 4 (Almost Always). Examples of CAMS-R questions include: “I am easily distracted.” “I am preoccupied by the past.” “I try to notice my thoughts without judging them.” “I can tolerate emotional pain.”

For a measure of trait affect, we used the Positive and Negative Affect Schedule, or PANAS (Watson et al., 1988). The PANAS consists of two scales, one for positive affect (PA) and the other for negative affect (NA). Internal consistency for the time scale that we used was “excellent” (PA alpha = 0.88; NA alpha = 0.87). The two PANAS scales demonstrated the strongest convergent/discriminant validity when the relationship between PA and NA was compared to multiple other similar measures. Correlations between PA and NA and measures of distress and psychopathology were strong for NA and comparatively weak (and inverse) for PA, demonstrating good external validity with the more concise PANAS scales. Each scale has 10 adjectives that are ranked on a 5-point Likert scale from 1 (Very Slightly) to 5 (Extremely). Examples of PA include “alert” and “inspired.” NA includes “upset” and “afraid.” There are a number of time frame options that may be used when providing answers. In order to identify trait affect, we used, “Indicate to what extent you generally feel this way, that is, how you feel on average.”

In some instances, participants provided two options on a single Likert scale item, i.e., two neighboring levels were indicated when only one was requested. Presumably, they could not decide between the two, or both in some way applied for them. In such instances, we resolved that ambiguity by using the score closest to the mean of the levels for that item.

A full copy of the questionnaire (Meditation Survey Questions.docx), a copy of the questionnaire data that was used for this paper (Survey vs. and Descriptive.xlsx), the selection of variables used for statistical analysis in R (Survey 1 data for R.csv), and the data file used for the imputation (Survey 1 for Impute.csv) may be found at https://osf.io/cnghp/.

### Meditation Practices

We use the word meditation according to a “method” definition (i.e., as a mental training technique and not a state; Nash and Newberg, 2013). Participants used multiple methods of meditation. In Ananda Marga, these methods are organized into “lessons,” and used to varying degrees depending on the individual. These lessons are largely consistent with yoga practice methods outlined by Patanjali. Patanjali, believed to have lived in India in the early centuries of the Common Era, organized yoga practices into “eight limbs,” often called “ashtanga yoga” (Larson, 2012). The first three (Yama and Niyama: moral codes; and asanas: physical postures/stretching exercises) were not part of the lessons (Anandamurti, 2010c), but were considered to be partly included in additional “Supplementary Practices” (see below) and not part of “meditation” in this study. The remaining five limbs were considered to be components of the meditative process (Pranayama: breath control; Pratyahara: sense withdrawal; Dharana: concentration upon physical ideation points; Dhyana: meditation/abstract contemplation; and Samadhi: absorption/one-pointed concentration) and were present to varying degrees in the different lessons of Ananda Marga (Anandamurti, 2010d). In Ananda Marga, the primary meditation practice (known as, “first lesson”) included concentrating on the “sound” and “meaning” of an internally repeated mantra, while focusing on a specific point of concentration. The mantras and points of concentration varied across individuals, although the general procedure using those components was the same. No attempt was made to distinguish time spent on different meditative practices.

To quantify the amount of meditation practiced by participants we asked participants to report: (1) “Current daily time (in minutes) typically spent meditating” (MDM), (2) “Number of years of Ananda Marga meditation” and “Number of years of other types of meditation” (both summed together to produce: YR), and (3) an estimate of lifetime hours (LTH) of meditation. For LTH, participants were asked to specify the number of years the MDM had been maintained. Following that, they were asked to divide their remaining prior lifetime meditation practice into as many as three different sets of years. For each set, they were asked to estimate the typical amount of daily meditation practice. LTH was calculated by multiplying the daily meditation practice (in minutes) for each set by 365 and then by the number of years in that set. All sets were summed together and the total was divided by 60, generating the resultant estimated LTH.

### Chakras

**Figure 1:**
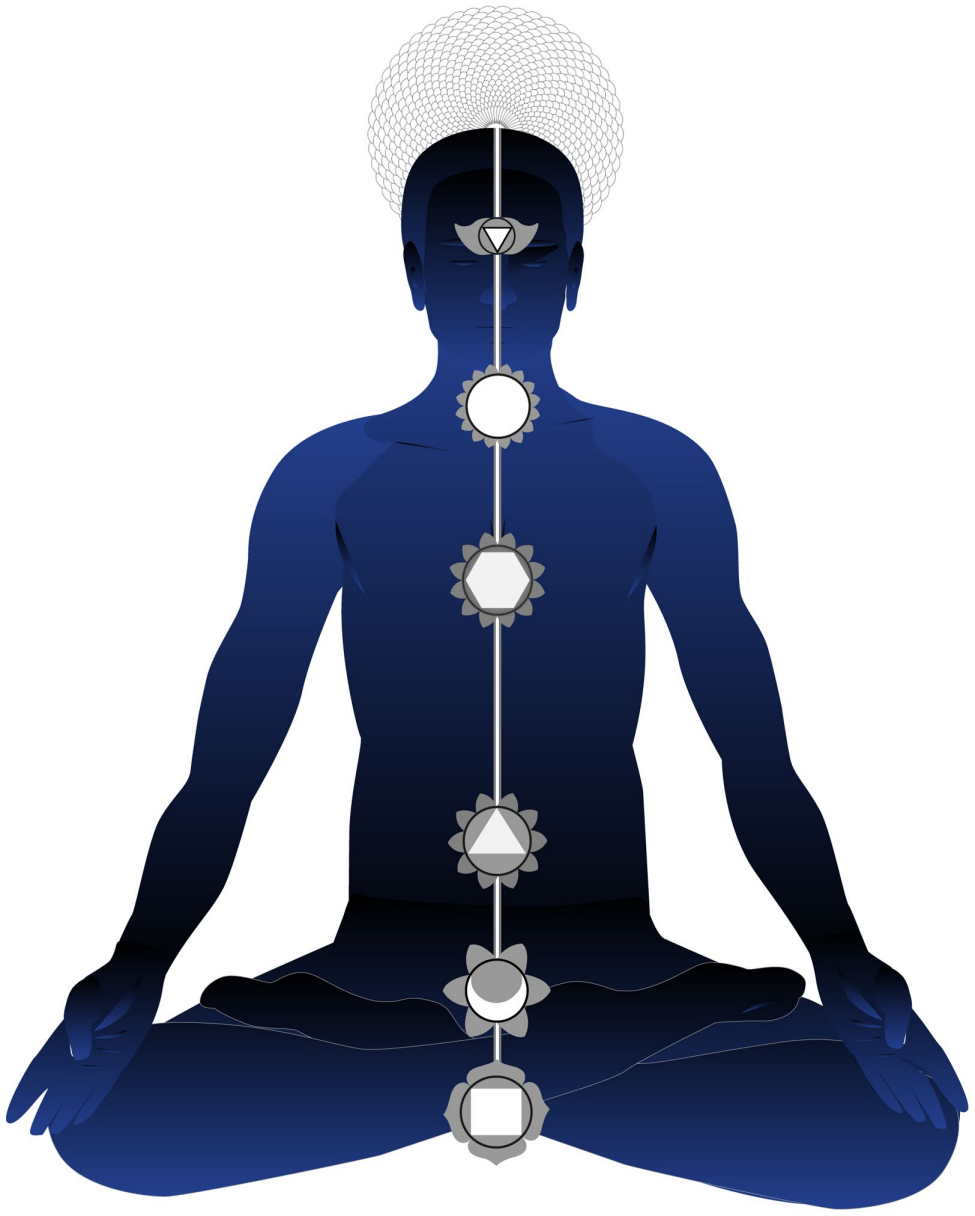
An artistic representation of the basic seven chakras used in Ananda Marga. Locations for the chakras are, respectively, in ascending order: the base of the spine above the perineum (*muladhara*, first chakra), at the genital organ (*svadisthana*, second chakra), at the navel (*manipura*, third chakra), at the midpoint of the chest (*anahata*, fourth chakra), at the throat (*vishuddha*, fifth chakra), between the eyebrows (*ajina*, sixth chakra), and at the crown of the head (*sahasrara*, seventh chakra). The locations of each chakra symbol represent concentration points on the ventral surface of the body, and are believed to reflect controlling points in the spine from where one can control certain mental dispositions (or *vrttis*) by influencing the secretion of glands in the vicinity of that point. The line connecting the chakras represents the *sushumna nadi*. Additional symbolic details are not presented here and are beyond the scope of the present discussion, including additional symbolic features related to the internal geometric shapes of each chakra, colors (presented in just neutral gray and white) and associated sounds. This digital artwork was created by Aaron Staengl and reprinted here with his permission.

Within Ananda Marga, a nine-chakra system is used, adding two chakras to the basic seven chakra system (Anandamurti, 1994). The basic seven chakra system (Avalon, 1974) is represented in Figure 1. The controlling points (*piitha* in Sanskrit) of the chakras are considered to be within the *sushumna nadi* which “passes through the length of the spinal column and extends up to the crown of the head” (Anandamurti, 2010b, p. 284). The concept of chakras is complex and, in addition to controlling points, includes concentration points and influence over glands within the region of each chakra. Chakra descriptions often include mixed portions of these three different aspects, but all are important and their physiological expression may originate from a common developmental process (Maxwell, 2009). The concentration points for each chakra are used for meditation practices and are located more ventrally than the controlling points. The concentration points of the chakras are at roughly the same vertical level as the controlling points. The locations for the concentration points include the base of the spine above the perineum (*muladhara*, first chakra), at the genital organ (*svadisthana*, second chakra), at the navel (*manipura*, third chakra), at the midpoint of the chest (*anahata*, fourth chakra), at the throat (*vishuddha*, fifth chakra), between the eyebrows (*ajina*, sixth chakra), and at the crown of the head (*sahasrara*, seventh chakra; Anandamurti, 2010b). For the additional two chakras, one is between the *vishuddha* and *ajina* chakras (*lalana* chakra), and the other is just beneath the crown of the head (*guru* chakra; Anandamurti, 2010a).

### Supplementary Practices

We calculated an additional composite variable that we called “Supplementary Practices” to identify the possible influence of supplemental practices on the quantity of meditation, and level of affect and mindfulness. Supplementary Practices included yoga asanas (yoga postures), diet, fasting, amount of sexual activity, and recreational drug use. Each of these was a dichotomous variable, or transformed into a dichotomous variable. Positive observance was given the value of “1.” The sum of these five scores produced the value of the Supplementary Practices variable. Additional explanation of each of the five practices may be found in the Supplementary Materials.

### Kundalini-Related Somatomotor Activity

We wanted to have a more specific measure of kundalini than the broad collection of signs and symptoms offered by Sannella (1992) and also described traditionally (Silburn, 1988; Feuerstein, 1997; Eliade, 2009; Anandamurti, 2020a,c). To do this, we calculated KRSM, an exploratory intra-subject dichotomous variable, based on the traditional description of kundalini as a rising power (Hatley, 2022) or force (Anandamurti, 1993a). All subjects who reported somatosensory or temperature sensations rising in the spine or back were included in KRSM (the back was included to cover less precise descriptions than the spine).

### Data Analysis

We used Cronbach alpha as a measure of reliability (Cronbach, 1951). As alpha is only valid for unidimensional data, we also tested the dimensionality of the data using DIMTEST in the EFA.dimensionality package in R. DIMTEST contains multiple indicators of dimensionality (including the Kaiser criterion, the empirical Kaiser test, traditional parallel analysis, comparison data test, the Hull method, sequential chi-square model test).

Additional explanation of the processes used for data analysis may be found in the Supplementary Materials.

## Results

### Incidence of Each Modality

**Figure 2:**
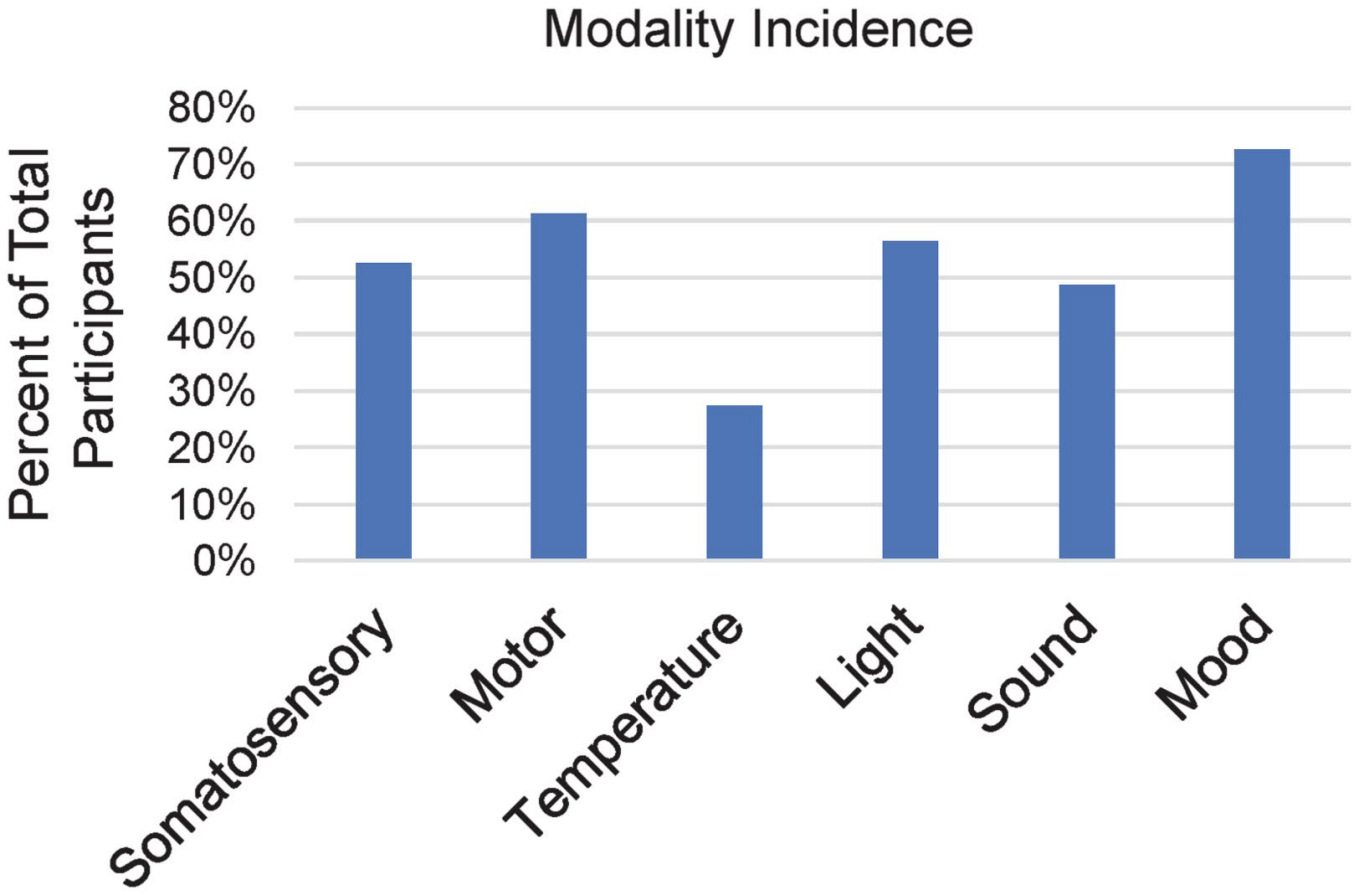
Bar chart for the percent of all participants (*n* = 80) who reported having experiences within any particular modality.

We first examined general relationships among the six modalities (Somatosensory, Motor, Temperature, Light, Sound, and Mood), beginning with how commonly each occurred during meditation practice for our participants. Figure 2 shows the percent of participants that reported a spontaneous experience in each sensory and motor modality or a shift in mood associated with meditation practice. The greatest number of participants reported experiencing a mood shift (*n* = 58, 73%). The next most common modality was motor experiences that involved some type of spontaneous physical activity (*n* = 49, 61%). This was followed by sensory experiences associated with light (*n* = 45, 56%), somatosensory (*n* = 42, 53%) and sound (*n* = 37, 46%). Experiences associated with a sense of change in temperature in some portion of or throughout the body occurred in the fewest number of participants (*n* = 22, 28%). Using logistic regressions for gender with each variable and linear regressions for age, no significant relationships were observed after correcting for multiple measurements with the false discovery rate procedure, “FDR,” (Benjamini and Hochberg, 1995).

Four participants (5%) reported having no experiences in any of the modalities. These four were not beginners but had diverse quantities of meditation ranging from approximately 5,200 to 33,000 LTH. Three participants (4%) reported experiences in all six modalities, ranging from about 3,300 to 16,400 LTH. No somatomotor (somatosensory, temperature and motor) experiences were reported by 20 participants (25%); no audiovisual (sound and light) experiences were reported by 24 participants (30%); and four participants (5%) reported only affective (mood shift) experiences. The remainder of the participants reported experiences in varying combinations of the modalities.

### Relationships Among Modalities

We next examined the relationships among the six modalities using a separate logistic regression for each modality in relation to the other modalities (Table 1). Somatosensory was significantly related to Motor (*z* = 3.160, *p* = 0.008; FDR-corrected) and there was a tendency toward significance with Temperature (*z* = 2.194; *p* = 0.071; FDR-corrected). However, when Temperature was the dependent variable, there were no significant relationships. Motor was significantly related only to Somatosensory (*z* = 3.194, *p* = 0.007; FDR-corrected). Sound and Light both had a tendency toward significance with each other (*z* = 2.51, *p* = 0.06; FDR-corrected). Mood had no significant relationship with any of the other modalities. For subsequent analyses, we organized the analysis of modality experiences according to these groupings: somatomotor (Somatosensory together with Motor and Temperature); audiovisual (Light with Sound); and affect (Mood).

**Table 1:** Modality logistic regressions.

	Somato-sensory	Motor	Temperature	Light	Sound	Mood
Somatosensory		3.160^**^	2.194.	1.066	0.799	0.957
Motor	3.194^**^		0.304	0.587	−0.126	1.521
Temperature	2.266	0.317		0.222	−0.017	−0.602
Light	1.113	0.625	0.214		2.513.	−0.419
Sound	0.933	−0.113	−0.119	2.510.		0.368
Mood	1.045	1.524	−0.598	−0.376	0.415	

Each row presents the *z*-scores from a logistic regression with one dependent variable (one modality) and five independent variables (the remaining modalities). The dependent variable is located in the first column. The remaining columns represent the independent variables. *N* = 80. All probabilities were corrected for multiple comparisons using the “FDR” approach. Significance codes: ‘ ’*p* < 1; ‘.’*p* < 0.1; ‘^**^’*p* < 0.01.

### Internal Reliability for Modalities

We assessed the dimensionality of the modalities using the DIMTEST procedure. We used six dimensionality indicators. All but one converged on a single factor, indicating that our modality data was unidimensional. Accordingly, we used Cronbach’s alpha to measure reliability (Cronbach, 1951). The alpha level we calculated for the six modalities was 0.572.

### Quantity of Meditation Practice

Participants’ mean minutes of daily meditation (MDM) was 86.2 (SD 46.3, range 10–225) and they had been meditating for a mean of 17.5 years (YR; SD 12.6, range 2–39). The mean for their lifetime hours of meditation (LTH) was 8,431 (SD 8,282, range 61–32,850). Within this sample, 32 participants (40%) had greater than 10,000 LTH. The logistic regressions for gender demonstrated no significant relationships with any of the meditation variables, or the Supplementary Practices variable. However, linear regressions demonstrated highly significant relationships of age with YR (*t* = 14.1, *p* < 0.001) and with LTH (*t* = 9.4, *p* < 0.001), an anticipated unavoidable severe bias for which we have used statistical correction.

### Individual Modalities

#### Somatosensory (Somatomotor)

**Figure 3:**
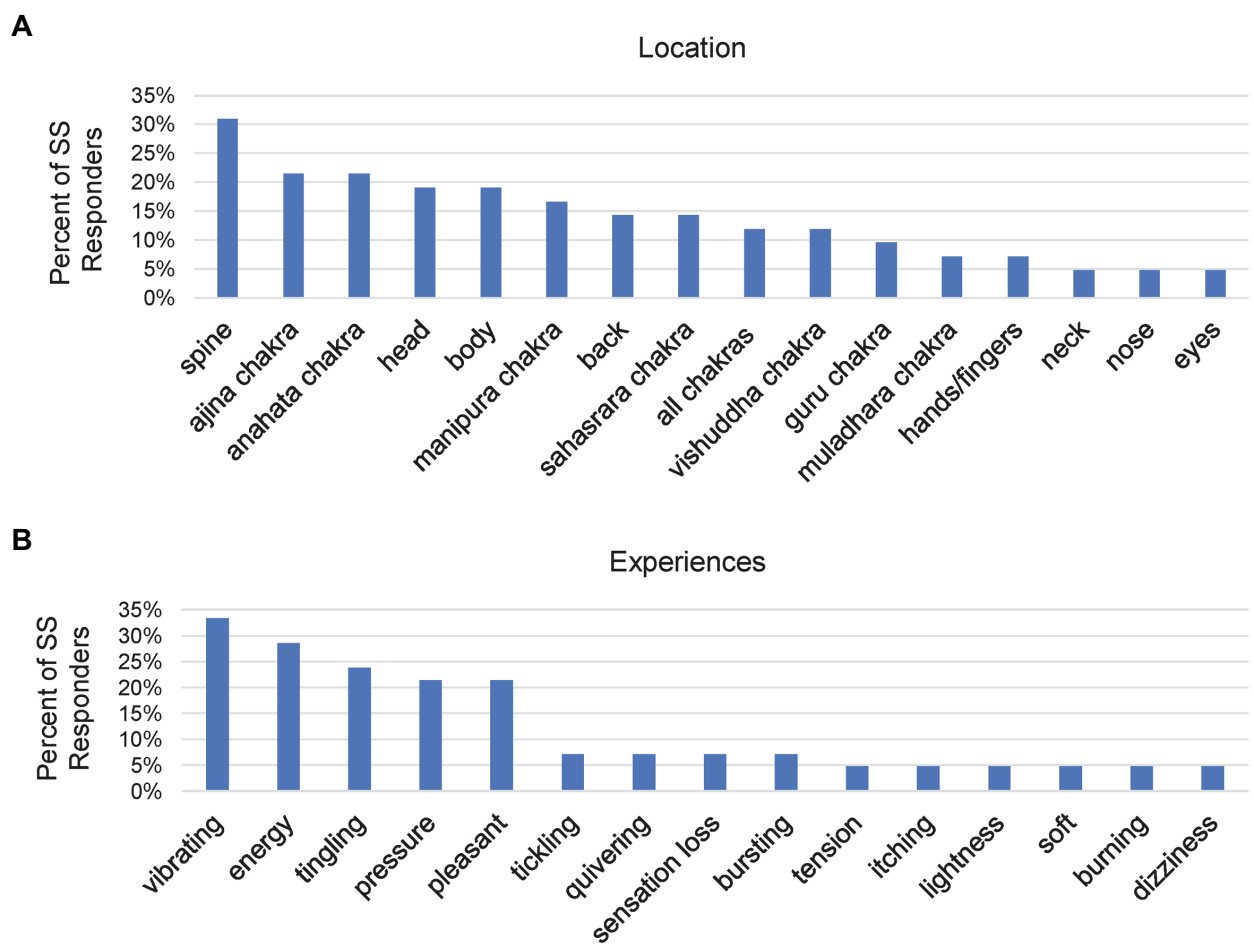
Bar chart for the percentage of Somatosensory (SS) responders who reported various somatosensory characteristics, including **(A)** various locations of somatosensory experiences, and **(B)** specific types of somatosensory experiences. Participants were allowed to report as many different locations and experiences as desired.

Since kundalini has been described as rising upward through the spine while piercing the various chakras (Anandamurti, 1993b), we were interested in how reports of spontaneous somatosensory experiences were localized at specific points on the body. Each participant could report as many locations for somatosensory experiences as desired. Within the 28 participants (67% of Somatosensory responders) who reported locations (Figure 3A), the spine had the greatest number of endorsements (*n* = 13, 31% of Somatosensory responders), followed by *ajina* chakra, and *anahata* chakra (*n* = 9, 21%-for each). Less common responses included body parts such as head, back or body, other chakras, or “all chakras.”

In addition to somatosensory experiences occurring at different points on the body, we were also interested in how participants qualitatively described somatosensory experiences which arose during meditation (Figure 3B). Participants were allowed to report as many different somatosensory experiences as they wanted. The most common ways participants qualified somatosensory experiences were by using the words, “vibrating” (*n* = 14, 33%), “energy” (*n* = 12, 29%), “tingling” (*n* = 10, 24%), “pressure” (*n* = 9, 21%), and “pleasant” (*n* = 9, 21%). There were also a large number of unique responses (*n* = 26; not shown in Figure 3B), demonstrating the diversity of how these experiences were described.

In addition to the descriptions of locations and qualities of somatosensory experiences, we also were interested to know if the sensations moved through different locations and how that was described. Sensations changed locations for 17 (40%) Somatosensory responders out of 27 who gave responses to that question. Of those who reported changing locations, 13 (31%) reported rising sensations in the spine or back (see Somatosensory responses in Table 2 for examples). In two instances, sensations were reported to travel both upward and downward (e.g., P-64 in Table 2). Other responses included movement in relation to changes in concentration points (which occur in some practices), pulsations in relation to the breath, expanding outward from a particular chakra, and involvement of the extremities or the face in energy movements.

**Table 2:** A sample of modality responses demonstrating potential signs of kundalini-related activity, with participant number (P-#), estimated number of lifetime hours of meditation (LTH), modality of experience being reported, and quotes of experience reports. In addition to putative kundalini-related somatosensory experiences, experiences from other modalities reported by the same participant are provided.

P-#	LTH	Modality	Sample kundalini-related experiences
P-19	17,885	Mood	Not sure if it is unusual-often experience a feeling of relaxation, letting go, surrender or peace. Sometimes spikes of devotional longing, deep absorption, love, or sense of grace
P-31	13,475	Somato sensory	Falling over, shaking; vibrating; along spine, hands; up kundalini to head
		Motor	Arms shake, breathe in quickly, grunt or say “Baba”
		Light	Moderately bright, white, circular, occasionally
		Mood	Samadhi, bliss
P-32	26,919	Somato sensory	Whole body becomes tense, vibration from the base of the spine up through the neck, quivering throughout the trunk, head and limbs; becomes quiet with intense meditation
		Motor	Jerking, rhythmic, spasmodic; often spontaneous vocalization “humm,” less if I am able to channel the energy; slow and deep [breathing]
		Light	Very bright, consistently, [duration] with focus, [color] depends
		Sound	Crickets consistently, hissing, roaring and ocean infrequently
		Mood	When I am tired, meditation re-energizes my mind. I always feel calm and more centered after meditation. I become less attached and am able to think more clearly
P-33	2,981	Somato sensory	Vibrating became very strong-like a strong pulsating wave traveling up my spine-I thought I might vomit
		Mood	Sometimes after deep meditation, I feel a deep peace. Mind is silent and still, with a soft loving feeling. But I have also felt negative emotions and images come up, as if a pot lid was being opened
P-48	1,460	Somato sensory	Waves of pleasure all over, along the spine
		Motor	Occasionally a sudden straightening of spine when meditating, small grunt, more like a humm, occasionally a slowing of the breath
		Mood	A feeling of aloofness
P-64	16,425	Somato sensory	Tinglings up and down the sushumna nadi [central spinal channel], very pleasant; in all chakras much energy and very nice feeling
		Temp.	Heat inside, infrequently, sometimes longer than 30 min
		Motor	Sometimes it shakes me lightly, jerky and also smooth, often I come automatically into ujjai [or ujjayi] breathing [nasal diaphragmatic breathing with a throat constriction that causes a sound like snoring or “heavy” breathing]
		Light	Very bright light, in the head, consistently during meditation; lasts secs to mins; I understood the whole universe and saw Akasha Chronicle
		Sound	Roaring and bells occasionally, Om infrequently, all only in meditation
		Mood	I’m much more calm

Nine participants (21%) described positive feelings associated with their somatosensory experiences (see P-64 in Table 2), while two (5%) referred to negative feelings (see P-33 in Table 2).

#### Motor (Somatomotor)

We suspected that spontaneous muscle activity may potentially provide clues to kundalini-related energy states within the body (Vishnu Tirtha, 2000). We were interested to identify muscle activity that was spontaneous and involuntary and experienced in relation to meditation. Participants were allowed to report as many motor experiences as they wanted. Table 2 provides a variety of examples.

**Figure 4:**
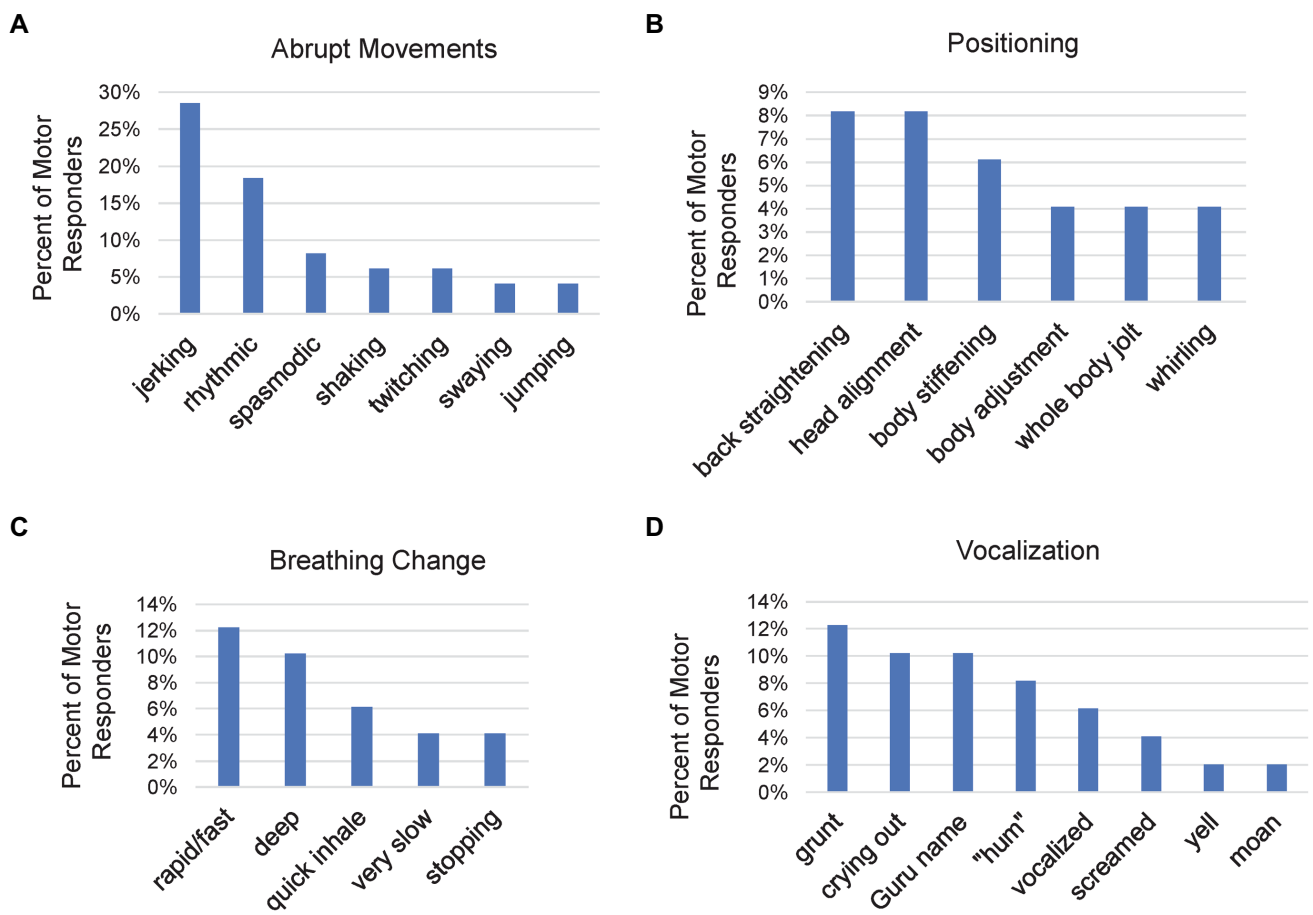
Bar charts of characteristics for different types of motor responses including, abrupt movements **(A)**, positioning movements **(B)**, breathing changes **(C)**, and vocalizations **(D)**. Each graph shows the percent of Motor responders which provided the various responses. Participants were allowed to report as many different experiences as desired for each bar chart.

Motor experiences were divided into abrupt movements, positioning movements, breathing changes and vocalizations. Abrupt movements were the most frequent type of motor event, occurring for 33 participants (67% of the Motor responders; Figure 4A). Participants qualified abrupt movements most commonly by using words like “jerking” (*n* = 14, 29%) and “rhythmic” (*n* = 9, 18%). Many response descriptions were unique, generated by only a single responder (*n* = 8; not included in Figure 4A), often implicating an energy release such as “violent,” “jolt” and “bouncing.”

The positioning category had responses from 26 participants (55% of motor responders). Positioning and the abrupt movement categories had 20 overlapping participants. The most mentioned positioning movements (Figure 4B) were back straightening and head alignment (*n* = 4, 8% for each), and body stiffening (*n* = 3, 6%). There was a higher occurrence of “unique” responses (*n* = 9) that were not included in Figure 4B. Examples were “back twist” and “hand configuration.”

The presence of “odd breathing patterns” (Figure 4C) was reported by 25 participants (51% of motor responders). “Rapid” or “fast” breathing occurred the most (*n* = 6, 13%), followed by “deep” breathing (*n* = 5, 10%) Also reported was “very slow” breathing (*n* = 2, 4%), “quick inhalation” (*n* = 3, 6%), and having breathing “stop” unintentionally (*n* = 2, 4%). The unique responses (*n* = 8) included energetic changes like “more intense,” “extreme energy” and “losing control” and were not included in Figure 4C.

Vocalizations involving an involuntary vocal expression of any form (Figure 4D) were reported by 23 participants (47% of motor responders). The most common were described as releasing a “grunt” (*n* = 6, 13%), “crying out” (*n* = 5, 10%), and calling out the word “Baba” (an affectionate name referring to the Guru; *n* = 5, 10%). One participant stated that “grunts” may be the same as the sound “hum,” (see P-48 in Table 2). Clarifying such relationships will require further research.

Some participants spontaneously reported associations between motor experiences and other modalities. P-32 described a rising vibration along with quivering, plus body jerking and consistent very bright light (Table 2). P-1 and P-69 in Table 3 provide additional examples of multi-modal complexity. Ten of the 13 participants who reported rising somatosensory experiences (not mentioning motor activity), also reported spontaneous physical motor movements; nine reported spontaneous vocalizations; and five reported breathing changes, all during meditation.

**Table 3:** Participants with unusually strong or unusual experiences (*) are presented with participant number (P-#), estimated number of lifetime hours of meditation (LTH), modalities of experiences being reported, together with quotes of experience reports. In addition to notable experiences, experiences in other modalities for the same participant are provided in order to have a broader context.

P-#	LTH	Modality	Anomalous experiences
P-1	1,399	Somato sensory*	If my meditation is intense, I will usually feel a lot of vibrating and energy-this usually occurs once a day, or every other day; rising up the spine, through the chakras
		Motor*	Jerky and spasmodic, but very rarely; the meditation can become too intense and noise comes out, very rare
		Light*	Extremely bright, all around me, as long as the intensity of the meditation; saw shining light around people several times. Twice I’ve seen visual spiritual messages appear in front of me
		Mood*	Meditation brings extreme bliss
P-14	23,634	Motor	Vibratory, mild shaking, subtle
		Light	Occasionally, bright, white, between my eyes (inside), briefly
		Mood*	Tremendous feelings of happiness-tears of joy-bliss
P-47	30,194	Motor*	When very concentrated, I sit straight without pain or effort for long periods-long deep breathing that is not ‘willed’ by me intentionally
		Light*	When visualizing light around someone expressing frustration/needing help, and taking the thought that help *could* come through me (from the spiritual world), I experienced waves of light going through me, forcing me to close my eyes and meditate, losing all awareness of the environment—The other person actually saw a vision of two saints appear where I was sitting, and stopped talking. He later said reading the works of those saints helped resolve his difficulty
		Mood*	Negative or reactionary mood becomes objective and joyful, forgiving, humble, honest, insightful. Lethargy or hopelessness, disinterest changes to enthusiasm, interest, wanting to help versus neediness
P-56	12,471	Somato sensory*	Monthly, upper body vibrates intensely, followed by a strong shaking experience
		Motor*	Doing pranayama, breath becomes extremely long and very deep and extremely intense
		Mood	In a good meditation, I sometimes feel very light, like I’m floating afterwards
P-69	4,015	Somato sensory*	Rising of kundalini beyond control-through all the chakras-to *sahasrara* -merging into white light
		Sound*	“wild wind”-I have to go through that to reach the light behind-2 to 3 times
		Light*	One time there was a point of white light first, then I merged; very bright, inexpressibly
		Mood	[nothing reported]
P-74	4,745	Light*	My whole body was made of light instead of flesh
		Mood	Relief, love

#### Temperature (Somatomotor)

We considered temperature experiences important to examine because of traditional literature associating heat with kundalini activity (Eliade, 2009). Participants were asked to specify temperature changes that were “independent of any medical condition or external source.”

Participants could report as many locations of the temperature experiences as they desired. The most common reports were that temperature changes occurred over the whole body (*n* = 11, 50%). Most of the remaining responses had unique locations (*n* = 10).

**Figure 5:**
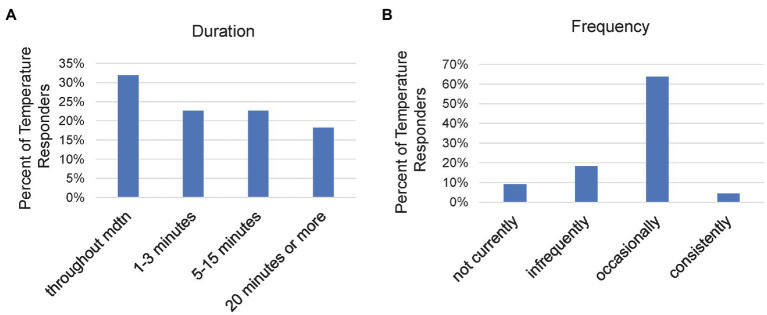
Bar chart for the percentage of Temperature responders who reported different Temperature characteristics, including **(A)** duration of temperature experiences, and **(B)** frequency of temperature experiences.

The duration of temperature changes was organized into four groups (Figure 5A). It was somewhat more common for the temperature change to last throughout meditation (*n* = 7, 32%). When “throughout meditation” (or a similar conditional phrase) was stated, the specific amount of time was unspecified. The remaining groups had similar incidence rates, short, 1–3 min durations (*n* = 5, 23%); intermediate, 5–15 min durations (*n* = 5, 23%); and longer, 20 min or more (*n* = 4, 18%).

The frequency at which temperature changes occurred for participants was also organized into four groups (Figure 5B). Occasional occurrence was the dominant frequency (*n* = 14, 64%). Consistent temperature change was rare (*n* = 1, 4.5%). Four subjects reported temperature experiences occurring infrequently (18%) and two had experienced them, but not currently (9%).

We also asked if participants experienced their temperature sensations moving across locations. Five of 20 participants indicated that the temperature sensations moved and two reported a clear upward movement including the back.

#### Light (Audiovisual)

While somatomotor experiences have been thought to distinguish the presence of kundalini-related activity (Corneille and Luke, 2021), light (effulgence or radiance), has also been considered a major kundalini-related expression of phenomenological significance in Tantric Yoga (Anandamurti, 2020a), as well as many other religious and spiritual traditions (Kapstein, 2004; Fox, 2008). We therefore looked at many aspects of light. Light was the sensory modality reported by the greatest number of participants.

In addition to the 45 participants who reported light experiences during meditation, three who reported no light experiences during meditation did have visual experiences that they associated with their meditation. One (P-43) saw “white light around things and people.” A second (P-47) saw light in “waves around me” and reported a complex experience in which she described a “healing” light moving through her and affecting a person with her who needed help (see Table 3 and the description in the “Anomalous Experiences” section). A third reported seeing an image of the Guru in a hallucination.

**Figure 6:**
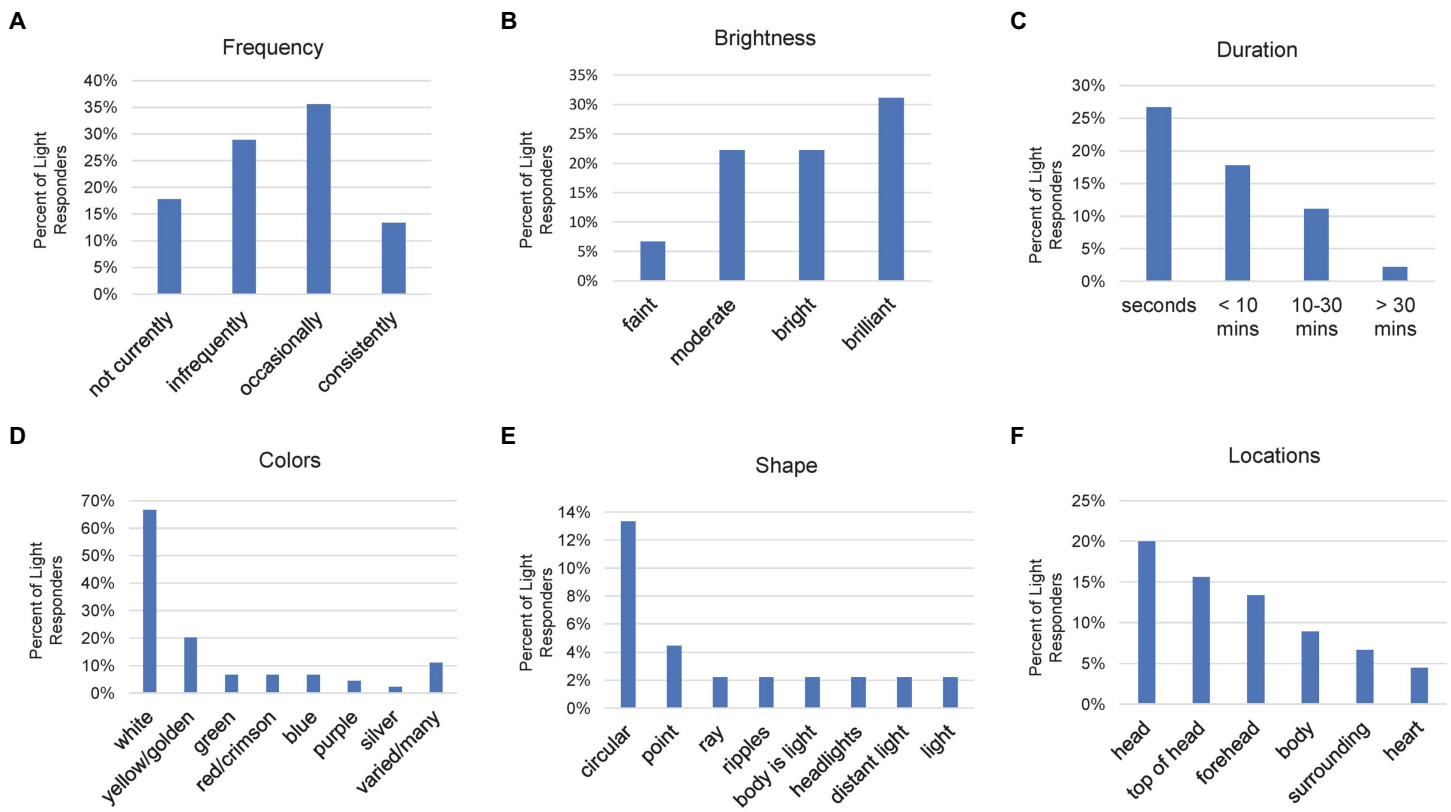
Bar charts of the percent of Light responders indicating various light characteristics: **(A)** frequency, **(B)** brightness, **(C)** duration, **(D)** colors, **(E)** shape, and **(F)** locations. Participants reported as many different experiences as desired for colors and locations.

Most of those reporting light experiences during meditation (43 of 45) provided a frequency from among four possible choices (Figure 6A). “Occasionally” (*n* = 16, 36%) was the most common and “consistently” was the least common, reported by 6 (13%). Of the six reporting “consistently,” only the two participants with the highest LTHs (both greater than 10,000 LTH) were in the KRSM (kundalini-related somatomotor) group.

In addition to the frequency of light experiences, intensity and duration are important characteristics. Intensity was defined by brightness, with 38 of the 45 light responders giving responses (Figure 6B). The highest level (“brilliant,” also including “very,” “extreme,” “max,” and “intense”) had the strongest endorsement (*n* = 14, 31%). “Moderate” and “bright” experiences occurred a similar amount (*n* = 10, 22% for each). Relatively few participants reported “faint” experiences of light (*n* = 3, 7%). The experience of brilliant light did not reliably predict KRSM membership. Half of those experiencing brilliant light were in the KRSM group, of which only three had an LTH over 10,000.

For duration, 27 gave responses (Figure 6C). The shortest time period (“seconds”) was the most common (*n* = 12, 27%), and only one indicated the longest time period, “>30 min” (2%). It was, however, unclear from the data whether participants who reported longer duration had such prolonged experiences during just one specific meditation practice or whether such experiences persisted beyond that particular practice.

We also examined other attributes of light like color, shape and location. By far, the most common color of light that participants experienced during their meditation was white (*n* = 30, 67%; Figure 6D). Five (11%) color reporters failed to name a specific color. Since 38 responses stated one or more specific colors, 79% of those naming a color, had a “white” response. “Yellow,” including “golden,” was the next most common (*n* = 9, 20%). Each participant could name as many colors as they desired. Six participants with yellow responses also gave a white response. The remaining responses were relatively uncommon, reported by only as many as three participants, for five additional colors. Some were also vague, simply stating “varied” or “many.”

Comparatively few responses (*n* = 15, 33%) were given for the shape of the light (Figure 6E). “Circular” (also including “sun,” “rings,” and “globe”) was the most common response (*n* = 6, 13%). Most of the remaining were unique responses. In contrast, 33 participants (69%) gave a response for the location or locations of their light experiences (Figure 6F). In the Somatosensory section, we tallied reports of physical locations and chakra locations separately because there was a greater diversity of responses which made relationships less clear. For Light, we have combined corresponding physical and chakra locations because without other competing options, the correspondence appeared logical. “Head” was reported most often (*n* = 9, 20%), followed by “top of head,” including *sahasrara* chakra (*n* = 7, 16%), and then “forehead,” including *ajina* chakra (*n* = 6, 13%). If all participants that referenced the head in some manner were combined, the head would be the primary location for light experiences (*n* = 21, 47%). Eliminating six vague or non-specific responses would give 21 of 27 participants (78%) reporting an area of the head as the location of their visual experiences.

#### Sound (Audiovisual)

Sound is another sensory modality that lacks an easily recognized relationship to kundalini-related energetic changes, although traditional literature has linked various sound experiences with the rising kundalini (Anandamurti, 1999; Mallinson and Singleton, 2017). Sound experiences during meditation were reported by 39 participants.

**Figure 7:**
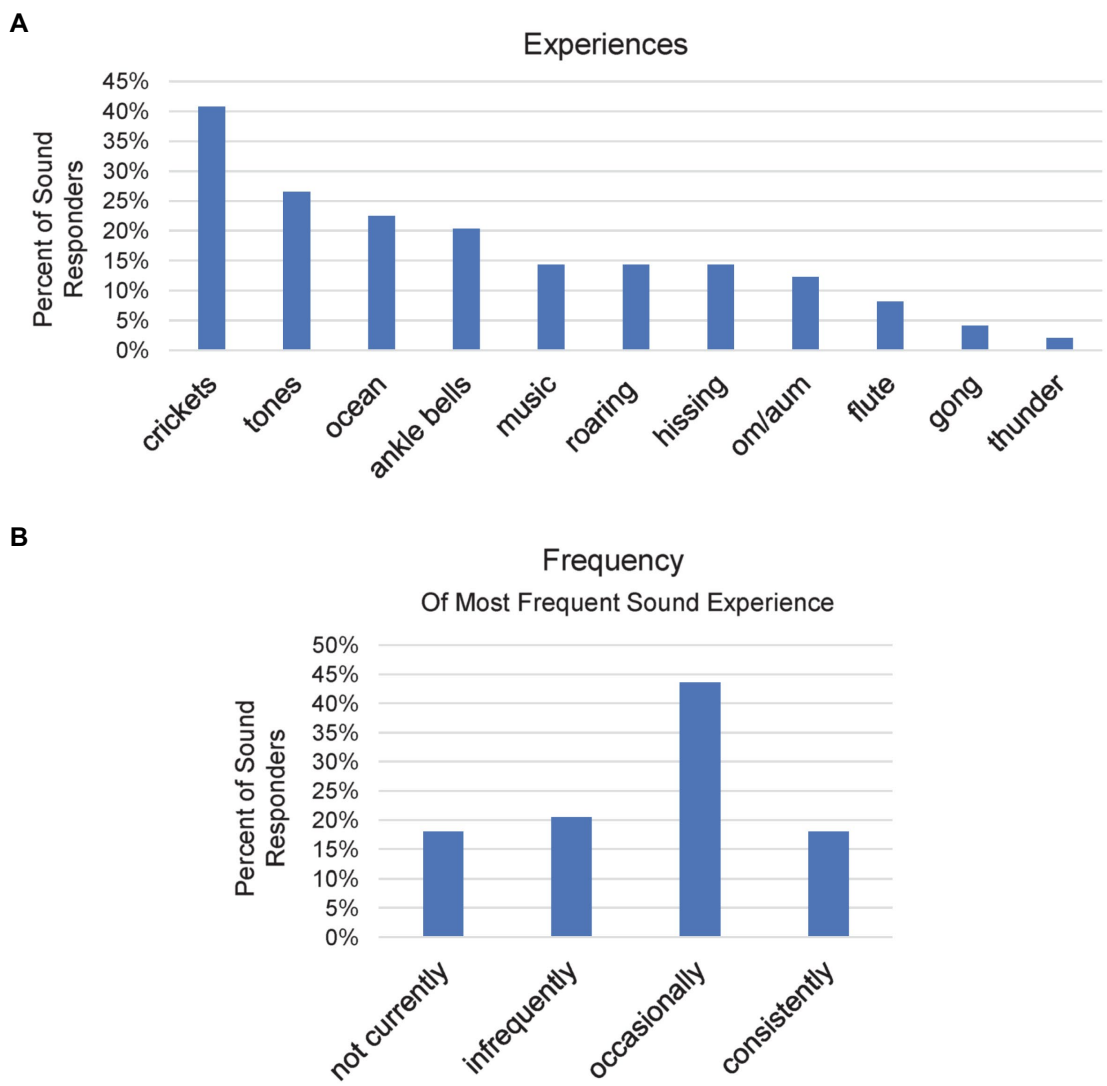
**(A)** Bar chart demonstrating the incidence of each sound experience occurring during meditation as the percent of total Sound responders. **(B)** Bar showing how frequently participants who reported Sound experiences had such experiences. Each participant is represented only once.

Of the various options, the greatest number of participants reported hearing the sound of “crickets” (*n* = 20, 41% of Sound responders) during meditation (Figure 7A). “Tones” (*n* = 13, 27%) were the next most common response, followed by the sound of the “ocean” (*n* = 11, 22%) and “ankle bells” (*n* = 10, 20%). Nine participants (18%) provided 10 independently specified responses (not included in Figure 7A). Three (6%) stated some type of “voice” experience (“inner voice,” “voices of spiritual guidance,” and “a voice coming from beyond). Others were fully unique, ranging from “a certain kind of hum,” “kirtan” (a kind of chanting), “indescribable,” and “sound stopped” to “the confluence of two waters” and “fine golden rain.”

Participants also specified the frequency of each of their sound responses. In order to have a general sense of how frequently participants had sound experiences, each participant’s greatest reported sound frequency was graphed. “Occasionally” (*n* = 17, 44%) was the most common (Figure 7B). The remaining frequencies had similar rates of occurrence. “Crickets” had the greatest number of “consistently” responses during meditation (*n* = 5, 13%). Six participants (15%) reported hearing the om/aum sound during meditation. No one experienced the om/aum sound “consistently.” P-43 was the only participant who reported experiencing om/aum “occasionally” when meditating. She had a relatively low LTH (365). One other om/aum reporter also had a low LTH (P-66; 183 LTH). However, other om/aum reporters had more meditation practice, ranging from 4,380 LTH to 16,547 LTH.

#### Mood (Affective)

We were interested to identify how affective feelings shifted with meditation. Of the 80 individuals in this study, 58 (73%) indicated that they had experienced an “unusual shift in mood” as a result of their meditation and other spiritual practices. Descriptions of this mood shift were quite varied. Of those who provided descriptions (*n* = 56), all but one response included positive elements (98%), or in relation to all 80 participants, 69%. Consistent with this, the vast majority of the words used had a positive connotation.

**Figure 8:**
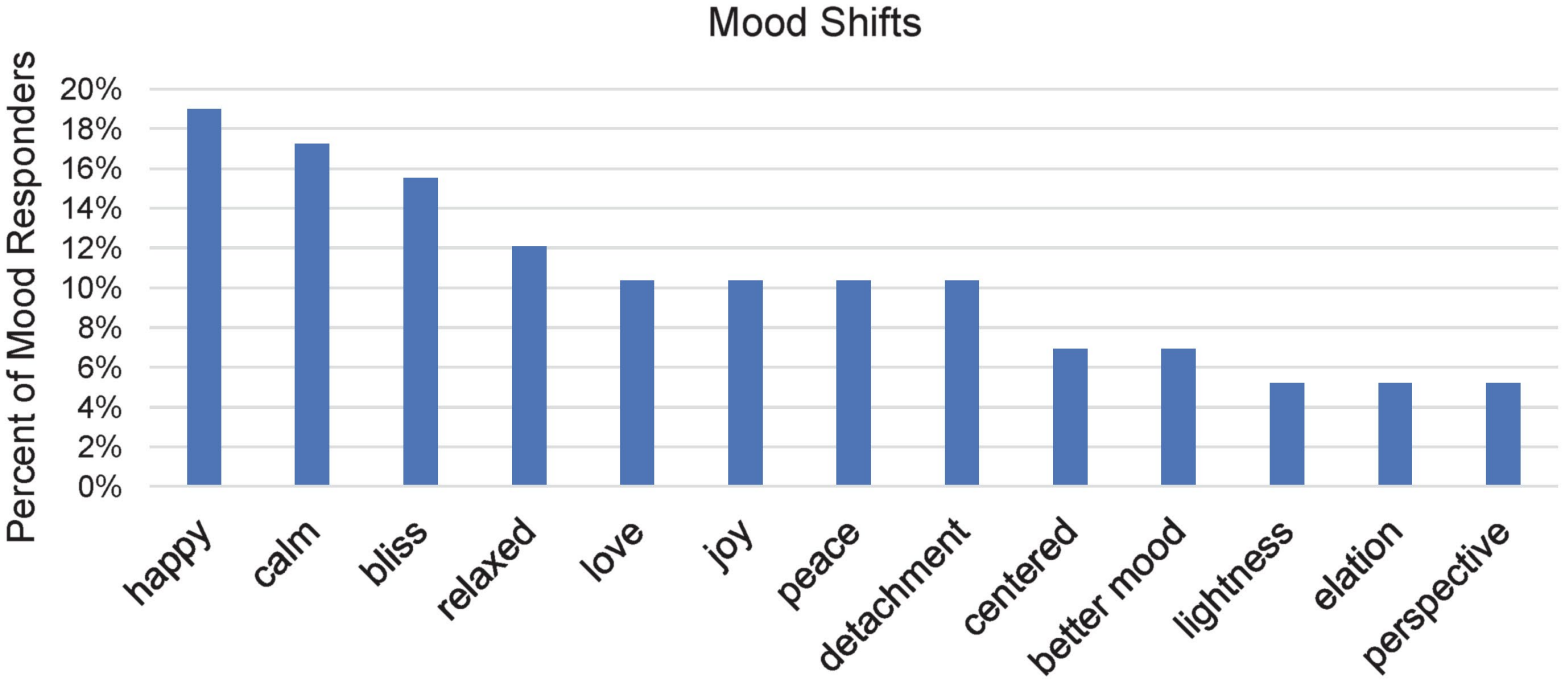
Bar chart demonstrating the percent of Mood responders reporting various mood shifts. Participants were allowed to report as many different types of mood shifts as desired.

The most frequent words used in these descriptions are shown in Figure 8. “Happy,” or grammatical variants, was the most common response, given by 11 participants (19% of those responding to the question of a mood shift). “Calm” was a close second (*n* = 10, 17%), followed by “bliss” (*n* = 9, 16%). “Relaxed,” “love,” “joy,” “peace,” and “detachment” were other comparatively frequent words. In addition to some less frequently used words, 20 words were used that were not used by anyone else. Not quite as many words, 13, were used by two participants (neither group was included in Figure 8). The instances of negative words were in these latter two groups.

Twice there was reference to “anger” arising, and a similar number for “sadness” and “tearing.” For a different participant, “tears of joy” was mentioned once. One vague reference was made to “negative emotions” occurring in meditation. In four out of five references to some form of negative emotions, positive experiences were described first. For example, P-24 stated, “Often meditation has a powerful uplifting effect on my mood, this was to be expected. However, at least once I became irritated after doing meditation. More than once, I have felt very sad, even moved to tears.”

### Relationships Among Modalities, Meditation Quantity, and Psychological Variables

In addition to the qualitative information we gathered, we were interested to identify in what way modality activity was related to four other variables (MDM, YR, LTH, and Supplementary Practices) using logistic regressions. Each logistic regression had one modality as the dependent variable and the four independent variables. No significant effects were observed.

We next used logistic regressions similarly to individually compare each of the modalities (dependent variables), to three different independent variables (mindfulness, PA, and NA). There were no significant relationships for any of these logistic regressions.

A final set of logistic regressions was conducted with the dichotomous variable “KRSM” (representing all participants with kundalini-related somatomotor activity, defined as somatosensory or temperature experiences of rising sensations in the spine and/or back). Fifteen participants (19% of all participants) met criteria for inclusion in KRSM. KRSM was used as the dependent variable with the six modalities as the independent variables. No significant relationships were found. Two additional logistic regressions were performed with KRSM as the dependent variable. When MDM, YR, LTH, and Supplementary Practices were the independent variables, no significant relationships were present. Similarly, when mindfulness, PA, and NA were the independent variables, there were also no significant relationships.

### Partial Correlations Among Meditation Quantity and Psychological Variables

Since strong correlations were present, as expected, for age with YR (*r* = 0.85, *p* < 0.0001) and LTH (*r* = 0.73, *p* < 0.0001), we used partial correlations removing the contribution of age. This allowed us to examine relationships independent of age between other variables, particularly in relation to quantity of meditation. Each partial correlation had significance adjusted for multiple measurements using an “FDR” adjustment. Strong partial correlations (Table 4) were observed between MDM and mindfulness (*r* = 0.448, *p* < 0.001). YR had a significant partial correlation with PA (*r* = 0.270, *p* < 0.05). LTH had significant partial correlations with mindfulness (*r* = 0.311, *p* < 0.05) and NA (*r* = −0.279, *p* < 0.05). The partial correlation with NA was inverse, meaning that higher negativity occurred for lower LTH.

**Table 4:** Partial correlation table for meditation and psychological variables.

	PA	NA	Mfl	MDM	YR	LTH
NA	−0.368^**^					
Mfl	0.402^***^	−0.513^***^				
MDM	0.199.	−0.173	0.448^***^			
YR	0.270^*^	−0.180	0.204.	0.166		
LTH	0.222.	−0.279^*^	0.311^*^	0.561^***^	0.674^***^	
Prac	0.247^*^	−0.141	0.297^*^	0.670^***^	0.217.	0.505^***^

Partial correlations for psychological variables, meditation and practices are listed. The effect of age has been removed. All significance levels have been adjusted for multiple comparisons using the “FDR” procedure. PA, PANAS positive affect; NA, PANAS negative affect; Mfl, mindfulness (CAMS-R Total Score); MDM, minutes of daily meditation; YR, total years of meditation; LTH, estimated lifetime hours of meditation; Prac, the composite of Supplementary Practices. Significance codes: ‘ ’*p* < 1; ‘.’*p* < 0.1; ‘^*^’*p* < 0.05; ‘^**^’*p* < 0.01; ‘^***^’*p* < 0.001.

The partial correlations table for meditation and psychological variables (Table 4) also included an exploratory Supplementary Practices measure (composed of five supplemental yogic practices). Supplementary Practices had highly significant partial correlations with MDM (*r* = 0.670, *p* < 0.001) and LTH (*r* = 0.505, *p* < 0.001), in addition to significant partial correlations with mindfulness (*r* = 0.297, *p* < 0.05) and PA (*r* = 0.247, *p* < 0.05).

Finally, there were highly significant partial correlations between LTH and MDM (*r* = 0.561, *p* < 0.001), and between LTH and YR (*r* = 0.674, *p* < 0.001).

Among the psychological variables, there were highly significant partial correlations for mindfulness, PA, and NA with each other in every combination. The partial correlations for NA with the others were negative, or inverse. See Table 4.

### Anomalous Experiences

Some experiences that were reported had more atypical characteristics, consistent with the interpretive and non-physiological (parapsychological or anomalous) categories of Sannella (1992). Some unusual light experiences occurred during meditation. When describing the form of light experienced during meditation, P-74 noted an experience in which her “whole body was made of light” (Table 3). Described under mood changes (not included in Table 3), P-84 reported that when he occasionally experienced very bright white light, “the breathing gets rapid and the heart beats fast,” and he had “great joy.” Elaborating further, he noted that, “After meditation, I feel tremendous love towards others.” P-69 (Table 3) reported a complex experience also involving multiple modalities. This experience included somatosensory feelings of kundalini uncontrollably rising and reaching the chakra at the top of her head. Associated with this, she experienced “inexpressibly” bright white light, into which she “merged.”

Additional unusual experiences with light not occurring during meditation (although considered related to the meditative practices) were described by a number of people. P-1 had “visual spiritual messages” appear before him and he also saw a “shining light” around people (Table 3). P-43 reported seeing “white light around things and people.” P-41 reported walking down a path together with others, but being the only one to see the path as lit. A total of 12 (27%) participants reported experiencing “abnormal environmental illumination.” Another 12 participants (out of which three overlapped with those experiencing abnormal environmental illumination) reported non-drug related visual hallucinations. Eight of those were spiritually-oriented hallucinations, seven associated with the Guru.

Many experiences were reported that included unusual somatosensory and motor intensity. P-56 described “intense” upper body vibrating and “extremely intense” deep breathing (Table 3). P-1 reported that with “intense meditation,” in addition to vibrating, energy and jerkiness, he may experience a vocal release (Table 3). Similar intensity may have been present for five participants that reported “crying out” and two that reported they “screamed” (see Figure 4D). P-31 (Table 2) described “falling over, shaking” and under Mood stated “samadhi, bliss.” P-33 had sufficiently strong vibrating moving up his spine that he thought he might vomit (Table 2).

Participants described many powerful positive, potentially ecstatic, experiences. Two participants of the nine that used the term “bliss” were included in Table 3, because of the intensity of their descriptions. P-14 reported minimal other experiences, but in addition to reporting “bliss,” he stated “tremendous feelings of happiness, tears of joy.” P-1 reported “extreme bliss” from meditating. A sample of other reports not included in Table 3 demonstrated other ways of expressing strong positive feelings: “waves of pleasure all over” (P-48); “Much calming. Deeply rooted joy.” (P-17); “Bliss! Quiet-no thought state.” (P-45); “feelings of elation” (P-21); and “intoxication” (P-16) which is a term often used to describe spiritual ecstasy (Anandamurti, 1982). While not as intensely positive, P-47 gave a rich description of positive changes under Mood in Table 3. The amount of meditation associated with these powerful positive experience descriptions covered virtually the whole range, from 593 to 22,634 LTH, not including P-47 who had more (30,194 LTH).

Additional reports included self-transformative experiences. In response to a question concerning having seen the environment illuminated by other than normal means, P-64 reported (Table 2) an experience in which she felt she understood the whole universe and saw the “Akasha Chronicle” [a concept within anthroposophy (Steiner, 1950) and also known as the Akashic Records in theosophy, representing a subtle compendium of all events in the universe]. Two others were not included in Tables 2 or 3. P-54 reported a “feeling of inner peace, messages flowing in mind, oneness with everything” in the context of the mood shift explanation. P-38 reported having out of body experiences in the context of breathing changes under Motor experiences (“sometimes I get out of the body experiences and my heart starts pounding and my breathing increases greatly in rate”).

The most notable spontaneous experience was reported by P-47 (Table 3), briefly mentioned in the Light section. As she visualized light surrounding a person sitting with her who was “expressing frustration/needing help,” she had the thought that help could come through her. She next experienced waves of light within herself and lost awareness of the environment, lapsing into meditation. She further reported that while she meditated, the person adjacent to her had a hallucinatory vision of two saints sitting in place of her. His later reading the works of those saints were claimed to have helped resolve the “difficulty” that he was experiencing. Given the highly unusual quality of this experience, it is unfortunate that greater details are not available. However, this was reported from a highly experienced participant who had the next to highest LTH. Nothing else in that participant’s responses suggested exaggeration or over-dramatization.

## Discussion

The present study investigated spontaneous experiences arising from a single system of meditation practice which occurred in multiple modalities including sensory, motor and affective qualities. Our sample was comparatively small (80) making it an exploratory pilot study, and all conclusions should be tempered with this understanding. The alpha level for the internal reliability of our modality data (0.572) was below levels considered “acceptable.” With our low sample size and few scales, a “fair” level of reliability should be 0.6 or higher (Pontekotto and Ruckdeschel, 2007). Our low alpha level may be due to variability in individual responses due to conditions such as environment/varying conditions in which the questionnaire was completed, fatigue/intolerance of the long questionnaire, and privacy issues/varying response to the personal nature of questions about their meditation. However, it has also been shown that low alpha levels can be due to too few items in a measure (Tavakol and Dennick, 2011). Given that our modality variables are each based on a single question, that limitation is probably present. Dimensionality and reliability matter when formal tests are being constructed, but that was not our intention. Therefore, the issue of reliability may not be relevant. Variability probably arose from the highly general nature of the initial questions that provided the data for the modality response frequencies. Our primary goal was to gather exploratory descriptive data concerning experiences arising during meditation. The value of what we have reported comes more from the descriptive details than from the dichotomous modality categories. On their own, the dichotomous modality data would be unlikely to generate a meaningful model of the richness of meditation experiences.

We first discuss the reported experiences within these modalities.

### Somatosensory (Somatomotor)

Since kundalini-related experiences are most commonly associated with somatic experiences (Corneille and Luke, 2021), we begin by analyzing somatosensory experiences. Prior studies associated with kundalini have not included information about chakras and their somatic locations. While the most common response in our study for the location of a somatosensory experience was the “spine,” two chakras were tied for second-most common location, namely *ajina* (between the eyebrows) and *anahata* (center of the chest). Many additional responses included references to other chakras. Bodily locations of chakras are used as concentration points in various ways in most Ananda Marga lessons. Overall, our results suggest that the spine and chakra points may be key somatic locations for spontaneous meditation experiences.

Research has shown that somatosensory experience can be influenced by attention to a particular body location, primarily promoting perceptions of “tingling,” “warmth,” and “numbness” (Tihanyi et al., 2018) which share some similarity to our most common Somatosensory reports of “vibrating,” “energy,” and “tingling.” Within the Tantric Yoga practices of our participants, attention during various practices was directed to specific physical locations, suggesting that directed attention could be a cause of some of our Somatosensory reports. However, directed attention may not explain some of the more complex somatosensory meditation experiences which include spontaneous experiences in additional modalities (for example, P-69 feeling “kundalini” uncontrollably rising to the chakra at the top of her head, and with this, experiencing inexpressibly bright white light, into which she “merged”).

Somatosensory sensations moving upwards in the spine or back are traditionally associated with kundalini (Anandamurti, 1993b; Goswami, 1999). These are consistent with prior clinical descriptions involving “moving energy” and “spine” (Sannella, 1992; Benning et al., 2018) and similar reports related to awakening experiences (Lockley, 2019; Woollacott et al., 2021), but potentially different in quantity. For example, the latter authors specifically sought participants who believed they had a “spiritual awakening.” They received reports of “tingling or creeping sensation” in relation to “energy rising up the spine” from 40% of all participants, and 65% reported “unusual flows of energy.” In another study examining sudden profound spiritual experiences (Corneille and Luke, 2021), 40% self-identified as having a “spontaneous kundalini awakening” rather than a “spontaneous spiritual awakening.” In contrast, only 19% of our participants (*n* = 15) reported either rising somatosensory or temperature experiences including the back and spine (representing the KRSM variable), but we did not require our participants to have had a “spiritual awakening” or “kundalini awakening” experience. At least one of “vibrating,” “energy,” and “tingling,” the most reported somatosensory sensations, were reported by 45% of our participants (without reference to “unusual flows”), which included all of the participants with rising spinal sensations. In a phenomenological study of multiple Buddhist meditation practices (Lindahl et al., 2017), 63% reported somatic energy of varied strength, sometimes also described as “surges.”

In addition to having different inclusion criteria, one possible explanation for our sample’s comparatively low incidence of rising somatosensory energy experiences, may be that some participants in our study lacked sufficient meditation practice to have successfully activated their kundalini. However, Lindahl et al. (2017) had a similar variability in meditation experience. Another possibility is that participants followed a general guideline to disregard transient experiential phenomena (Anandamurti, 1992). A third possibility is that the meditative process included a sensory withdrawal step where participants were taught to withdraw their attention from both embedded and embodied factors that might distract them from the instructed meditation method (Anandamurti, 2010c; Hewitson, 2014). If sensory withdrawal was completed successfully, somatomotor experiences would not reach conscious awareness. P-14 and P-47 (Table 3) were possible examples of this. They both had high LTHs and did not report any somatosensory or temperature sensations, also having only subtle motor experiences, but having strong positive mood shifts. Therefore, it may not be possible to infer the lack of rising energy from the absence of the reported experience of energy phenomena. This demonstrates the potential importance of identifying a reliable objective measure in future research.

Many medical conditions can create moving somatic sensations called “formication,” which is generally perceived as insects crawling either on or under the skin. These include psychiatric delusions, menopause, hepatitis, HIV, thyroid disease, anemia, neurologic dysfunction, drug side-effects, drug/alcohol withdrawal, and more (Koh et al., 2011; Cernovsky et al., 2021). Of the 15 participants included in KRSM, only 3 (20%) reported receiving a medical treatment. Two were women (49 and 77 years old), treated, respectively, for a skin condition and a thyroid disorder. The male (57 years old) was treated for a cardiovascular condition. Even if all three were considered to be formicating for a medical reason, it is unlikely formication was present for the rest. Our data suggests that spontaneous moving sensations experienced during meditation may lack a purely bottom-up sensory, top-down attentional or pathological explanation.

### Motor (Somatomotor)

Many varieties of motor activity have been associated with kundalini (Sannella, 1992; Greyson, 1993a; Vishnu Tirtha, 2000; Sanches and Daniels, 2008). A study of Buddhist practitioners (Lindahl et al., 2017) associated involuntary body movements with kundalini-like “surges of somatic energy.” We examined abrupt physical movements, positioning movements, vocalizations and breathing changes. Our participants reported roughly similar incidence of these motor experiences (29–41% of all participants, 61% having at least one type) to other studies that include 66% for bodily “manifestations” (Poloma and Hoelter, 1998); 37% for involuntary movements (Lindahl et al., 2017), and 48% for “rushes shaking the body” (Woollacott et al., 2021).

Medical causes for spontaneous movements such as Tourette syndrome or myoclonus could be posited, but are unlikely. Tourette syndrome has been shown to have a prevalence of about 0.01% (Levine et al., 2019) and myoclonus was lower (Eberhardt and Topka, 2017). Since extreme psychiatric illness was not reported and would likely have interfered with completing the questionnaire, a kundalini-related process could potentially better explain the presence of these spontaneous motor experiences.

Some specific motor characteristics have been associated with kundalini-related involvement. P-47, who had not reported rising sensations in her back and was not included in KRSM, reported that when she was “very concentrated” in her meditation, she could sit straight effortlessly and would experience spontaneous deep breathing. Maintaining a straight posture while meditating is commonly recommended, but when occurring spontaneously, without effort, it has been considered characteristic of an active kundalini (Vishnu Tirtha, 2000). If this relationship is true, it suggests that kundalini can be active without the experience of rising sensations. In addition, effortless deep breathing implies parasympathetic activity (Jerath and Barnes, 2009), but being “very concentrated” implies strongly focused attention, arousal, and sympathetic activity (Britton et al., 2014; Lumma et al., 2015). The simultaneous presence of parasympathetic and sympathetic activation is a known pattern of autonomic activity (Hugdahl, 1996) and has been considered a potential component of some spiritual altered states of consciousness (ASC; Newberg and Yaden, 2018). Autonomic coactivation could contribute to some kundalini-related experiences.

Other participants had contrasting autonomic involvement. Nine reported unusual slowing or deepening of their breathing, suggesting greater parasympathetic activity, a common observation in meditation studies (Benson et al., 1974; Tang et al., 2009; Olex et al., 2013; Katyal and Goldin, 2021). Nine other participants experienced rapid breathing or abrupt inhalation, sometimes in conjunction with an abrupt movement, suggestive of sympathetic activity (Hageman et al., 2008). Such experiences may include other modalities. For example, P-84 experienced rapid breathing and heartbeat at the same time as occasionally experiencing a very bright white light, all presumably sympathetically driven. These different breathing patterns were all included within the kundalini signs and symptoms identified by Sannella (1992) and imply kundalini activity may include a complex mix of autonomic influences and not just sympathetic and parasympathetic co-activation.

We found that somatosensory experiences were significantly related to motor experiences, with a trend toward a significant relationship with temperature experiences. Aspects of these three modalities have been described as related to kundalini activity. According to traditional explanations [e.g., Hatha-Yoga-Pradipika (Avalon, 1974)], motor experiences have been considered to result from the kundalini attempting to move through three blockages, or “knots” (*granthis*), in the sushumna channel. The ancient Yoga-Shika-Upanishad, thought to be composed *circa* 100 BCE to 300 CE (Flood, 1996), provides an analogy. It compares the process of piercing the knots by the upward rising kundalini with “piercing the joints of a bamboo stick by means of a heated iron rod” (Feuerstein, 1997, p. 110). Sannella (1992) contended that “kundalini can be blocked anywhere along its upward trajectory.” According to Anandamurti (1993b, p. 130) the chakras are points of “fetters” that “the spiritual aspirant has to snap” and that the “kundalini has to pierce.”

### Temperature (Somatomotor)

Temperature change, while the least common modality for our participants, was primarily experienced as elevated heat. The generation of body heat is mediated by sympathetic processes and typically controlled cortically (Charkoudian, 2003; Amihai and Kozhevnikov, 2015). There are a variety of medical conditions that can cause excess body heat, but medical causes are considered unlikely, because most temperature experiences for our participants arose during meditation and lasted no longer than the meditative period.

Our reported temperature experiences differed qualitatively from a historical description of such experiences related to kundalini and meditation (Avalon, 1974, p. 241–242). Avalon (a.k.a. Sir John Woodroffe) reported, apparently from direct observation, that “intense heat” is experienced from an activated kundalini, with lower sections of the body becoming cold as the kundalini moves higher, ultimately leaving heat present only at the crown of the head. None of our participants reported this pattern of activity. Other research (Corneille and Luke, 2021) found that three KAS Physical symptoms questions referring to “unusual cold” in the body were among the least likely experiences to be reported on the KAS. As Woodroffe’s description was not from empirical investigation, his claims seem to not be supported by current empirical data.

A recent study of individuals who had experienced “kundalini awakenings” (Woollacott et al., 2021) reported that energy rising up the spine was associated with “intense heat/burning pain” in 20% of their participants. However, none of our participants reported “intense heat” or “burning pain” (although, we did not specifically request that information).

### Light (Audiovisual)

Multiple examples of light experiences that have been associated with kundalini are easily identified in traditional literature, such as from the Pashupata-Brahmana-Upanishad and Chandogya Upanishad (Feuerstein, 1997), by Kashmiri Hindu Tantric Abhinavagupta (Silburn, 1988; Muller-Ortega, 2004), and more current writings (Anandamurti, 1968; Krishna, 1993; St. Romain, 2017). Therefore, we were interested in descriptions of light experiences and their relationships with other experiences.

Under “Quality of energy rising up the spine,” in the study by Woollacott et al. (2021), 18% reported experiencing “Brilliant light.” Light that was “brilliant” in our study was reported by the same proportion of all participants, and it was the most common light intensity. Only half of those participants reported experiencing rising sensations in the spine or back, and only one reported both as part of the same experience (although descriptions combining modalities had not been requested). Similarly, only a third of participants experiencing consistent light experiences reported rising sensations and, while not requested, no one described a direct relationship between them. Thus, for our participants, more powerful visual experiences were not reliably associated with rising sensations.

A spontaneous visual experience without an external source is normally considered a hallucination (Waters et al., 2021). Sensory deprivation and perceptual isolation have been shown to induce spontaneous visual hallucinations (Merabet et al., 2004; Lloyd et al., 2012). In a Buddhist meditation study, “hallucinatory” visual experiences were associated by some participants with the degree of concentration that they had achieved (Lindahl et al., 2014). It was proposed that a possible mechanistic explanation of visual hallucinations during meditation is that increased cortical inhibition arises from greater mental concentration, consequently increasing neuronal excitability in inhibited areas. Fluctuating concentration could explain the variable quality of our participants’ most common visual experiences which were primarily white, brief (“seconds”) and “occasionally” experienced.

Imagination, repeated prayer practice, and cultural support have been proposed to promote spiritual hallucinatory experiences (e.g., seeing visions, hearing the voice of God; Luhrmann and Morgain, 2012). Such a process, however, does not explain spontaneous spiritual experiences different from what practitioners imagine or focus upon. Twelve of our participants reported non-drug related visual hallucinations. Eight of those were spiritually-oriented, of which seven were associated with the Guru. It is possible that imagination, repeated practice and cultural support could have promoted some of the visual hallucinations reported to us. One additional complex visual experience unlikely to be related to these mechanisms (P-47 in Table 3) will be discussed in the Anomalous Experiences section.

As a whole, we were unable to identify a consistent relationship between visual experiences and rising somatomotor experiences. While Sannella (1992) included all of the visual experiences reported here within his kundalini-related experiences, our findings contain only one direct report (P-69) of a visual experience associated with rising sensations. This difference could be due, as suggested in the section on motor experiences, to kundalini-related activity that is not associated with noticeable rising sensations, and the fact that we did not specifically ask about which modality experiences occurred together with others.

### Sound (Audiovisual)

Sensory deprivation for light experiences (Lindahl et al., 2014) was described as potentially causing “hallucinations” from cortical inhibition. This could also apply for sound. Culturally supported expectations could promote misattributions in which a desirable sound is identified when it was actually something else (Luhrmann and Morgain, 2012), which was suspected in at least one instance. Other factors, such as undiagnosed tinnitus (Henry et al., 2014), Musical Ear Syndrome (Low et al., 2013) and the more common “earworms” or “stuck song syndrome” (Beaman, 2018) could be responsible for some sound experiences. However, these types of factors do not appear to account for the full range of sounds reported by our participants.

Anandamurti (1999) proposed that meditators might hear a progressive series of sounds (first crickets, followed by ankle bells, sweet flute, gong or ocean, and finally om/aum) as they deepened their meditation and kundalini rose higher through the chakras. Among our participants, cricket sounds were reported most, which might be expected for the first sound in the sequence, since sounds associated with higher chakras would have had the kundalini also pass through the lowest chakra, with its associated cricket sound. The incidence of additional sound experiences did not follow the order of the proposed sequence. If there is relevance to this sound sequence, it is not discernable with the current available information and requires further exploration. It may be that incidence is not a good metric with which to identify this sequence.

It has been demonstrated that mental state changes, such as hallucinations induced by perceptual deprivation (Pütz et al., 2006) and induction of inner light/energy in Zen meditation (Lo et al., 2003) can be distinguished by changes in alpha EEG through using button presses to mark the experience onset. This technique could be a means of discriminating various meditation induced experiential states (Lindahl et al., 2014). This could provide an objective approach that may be useful for increasing knowledge concerning spontaneous auditory and other sensory experiences during meditation, as well as experiences involving rising sensations that suggest kundalini.

### Mood (Affective)

Subjective experiences resulting from Yogic meditation have often been found to have a positive valence (Menezes et al., 2015; Patel et al., 2018; Katyal et al., 2020; Park et al., 2020). We found a high incidence of positive mood shift (69% of participants), similar to 75% for reports of positive affect among Buddhist meditators (Lindahl et al., 2017), and 70% for reports collected from the Religious Experiences Research Center archives at the University of Wales (Lockley, 2019), and higher than 32% for “mood and energy swing” in a group with “kundalini awakening” (Woollacott et al., 2021) and 46% for “positive affective states” from people reporting “awakening experiences” (Taylor and Egete-Szabo, 2017). This indicates our participants had a comparatively strong positive response to their meditation practices.

Of the 15 participants who reported rising energy sensations (KRSM), only two were in a group of participants (*n* = 14) who had used stronger positive terms (e.g., bliss, tremendous or unbounded happiness, elation, great joy, and exalted love) to describe the positive affective shifts arising from their meditation. This demonstrates another instance in which our KRSM kundalini measure based solely on rising somatomotor experiences fails to distinguish a feature (unusual or extreme emotion) that has been associated with kundalini (Sannella, 1992).

Adverse meditation events have also been examined recently (Cebolla et al., 2017; Anderson et al., 2019; Farias et al., 2020; Goldberg et al., 2021; Lambert et al., 2021). In particular, Farias et al. (2020) found an overall prevalence of 33% for adverse meditation events in observational studies. In the present study, the valence of a mood shift was identified from response explanations. We found only 6% reported any negative effect of their meditation on mood. The failure to specifically ask about positive and negative experiences might have promoted underreporting of undesirable outcomes. We also did not include measures that could have identified clinical issues that may have been present, as indicated in some prior studies (Persinger, 1993; Thalbourne and Fox, 1999; Antonova et al., 2016), or in conditions of kundalini syndrome (Sannella, 1992; Greyson, 1993b; Valanciute and Thampy, 2011; Benning et al., 2018). We did request reports of any treatments being received and only one participant reported receiving any medical treatment for a mood disorder. The lower incidence of negative experiences is consistent with our expectations and could demonstrate that the kundalini “awakening” within our sample may have been more effectively balanced through the comprehensive Tantric Yoga practices and proper guidance than that experienced in circumstances of kundalini “syndrome” (Suchandra et al., 2021).

### Meditation and Psychological Measures

We had predicted, based on prior research (de Castro, 2015), that there would be significant relationships between the incidence of modalities and the amount of daily meditation (MDM). Contrary to expectations, there was a lack of significant relationships between the modalities and any of the quantity of meditation measures. This implies that experiences occurring during meditation may be independent of the quantity of meditation practice.

Moreover, contrary to expectations based on traditional literature concerning kundalini (Feuerstein, 1997; Anandamurti, 2020a,c), our KRSM variable demonstrated no significant relationship with any of the modalities, using logistic regressions. This suggests that KRSM does not meaningfully capture any kundalini-related expression present within the six modalities. KRSM also had no significant relationship with any meditation quantity measure or the Supplementary Practices variable. This may indicate that individuals move through the meditation process on differing experiential paths, possibly due to differing proclivities when starting such a process. That kundalini-related experiences are highly variable appears consistent with the perspective that the way kundalini is expressed “is different for each individual” (Sarkar, 1978, p. 74).

There were also no significant relationships between any of the modalities and any of the trait measures (mindfulness, PA, or NA). KRSM also had no significant relationship with any of the trait variables. Thus, at least for this particular form of meditation, spontaneous experiences appear to lack any significance in relation to quantity of practices performed and psychological traits achieved. While some auditory and somatosensory experiences may signify progress in elevating the kundalini (Anandamurti, 1999), we have observed no indication that sensory experiences have any positive (or negative) associations. Cautions concerning caring about subjective experiences are present in some Buddhist meditation traditions, although there are differences of opinion: some considering meditation experiences to be signs of progress, and others considering them potential hindrances (Lindahl et al., 2017). Tantric Yoga literature also includes cautions that interest in specific experiences or abilities could potentially be contrary to the deepening of meditation (Anandamurti, 1992).

In contrast to the modality results showing no significant relationships, there were significant partial correlations (removing the age effect) for all three measures of the quantity of meditation practice with some of the trait measures, indicating psychological benefits occurred from the practice of meditation. This is consistent with our expectations based on prior research that showed greater years of meditation (YR) was associated with trait positive affect (PA; Easterlin and Cardeña, 1998), and LTH was associated with mindfulness and inversely with negative affect (NA). Also, consistent with expectations, quantity of daily meditation (MDM) was associated with increased mindfulness (de Castro, 2015). A relationship with kundalini expression could not be verified.

There was an additional strong partial correlation of MDM and LTH with Supplementary Practices. Supplementary Practices also had significant relationships with trait mindfulness and PA. Since LTH was based on both MDM and YR, and YR was not correlated with Supplementary Practices, individuals who put more time into their daily meditation may be more likely to observe more supplemental yogic practices. This probably represents a greater investment in the broader Tantric Yoga lifestyle. However, that does not appear to be the case for individuals with just greater YR.

### Anomalous Experiences

While we systematically examined spontaneous meditation experiences in six modalities, qualitative reports also suggested consequences of spontaneous experiences outside these modalities. Changes in self-awareness are a fundamental part of deepening meditation and elevating kundalini (Anandamurti, 1982). We received one report (P-69) of an experience of merging into light while losing a sense of individual self-awareness and three others that reported experiences with a universal quality or oneness. Highly positive, emotional states resembling ecstasy were reported by 14 participants. One participant reported out-of-body experiences, seven reported “visions” of the Guru, 12 experienced “abnormal environmental illumination” and three heard spiritual voices. Of these multiple participants, only one who heard spiritual voices was receiving any medical mental health treatment. Physiological and psychological explanations that we have described may have accounted for some of the experiences, but we have also noted limitations in those explanations. Other reports of anomalous phenomena, such as psychic abilities and the capacity to heal, have been identified in kundalini awakening research (Woollacott et al., 2021), but despite consistency with historical spiritual literature, mechanisms of such experiences are currently unclear.

A variety of complex brain mechanisms have been proposed to explain ASCs, including neurophysiological (Wettach, 2000; Bünning and Blanke, 2005; de Ridder et al., 2007; Borjigin et al., 2013; Gschwind and Picard, 2016; Newberg and Yaden, 2018) and neuropharmacological mechanisms (Barker et al., 2013; Newberg et al., 2017; Nichols, 2018; Dean et al., 2019). Additionally, an expanded conception of consciousness has been proposed as potentially necessary to explain ASCs, particularly ones that lack adequate neurophysiological or neuropharmacological explanation (Parnia and Fenwick, 2002; Baruss and Mossbridge, 2017; Woollacott, 2020). It has also been noted that even when valid neurophysiological and neuropharmacological explanations are present, they are correlational and do not demonstrate causality, leaving open the possibility that experiences may originate from other possible influences and be expressed through known mechanisms (Beauregard, 2020; Moreira-Almeida, 2020).

Within our data, a relevant example would be P-69’s experience of “kundalini” rising through the chakras beyond her control, culminating in merger of her self-awareness into a point of white light. This could be interpreted physiologically, such as through achieving a strong concentration which promoted cortical inhibitory mechanisms, or “deafferentation” (potentially parietal and occipital), together with including a preceding autonomic arousal to account for the rising sensations (Newberg and Yaden, 2018). While conceivable, it is unclear how completely this set of mechanisms would explain her full experience. P-69’s experience is consistent with traditional Tantric Yoga concepts of a rising kundalini and the presence of light as a quality of a universally pervading consciousness (Anandamurti, 2020b). Both of these explanations could apply, as suggested earlier. Another example is P-47’s visual experience and the second-hand report of the other person’s related experience (Table 3). If fully accurate, they represent a complex shared ASC that would be difficult to explain by known physiological mechanisms. We consider it valuable to recognize the presence of anomalous experiences and note their potential importance, even if we cannot presently explain them. Further work is required to distinguish the relative contributions of these different influences.

### Kundalini

Our primary marker of kundalini-related activity (rising energy together with somatomotor experiences: KRSM) was unrelated to any of our other measures. However, the consistency of our findings with Sanella’s broad analysis supports the contention that much of our findings might include kundalini-related influences which affect more than somatomotor experiences. Corneille and Luke (2021) observed that greater somatomotor experiences distinguished a group self-designating as having kundalini spiritual awakenings in comparison to a similar group that did not associate kundalini with their awakening experiences. However, Corneille and Luke’s overall total scores for the KAS, did not differ for the kundalini and non-kundalini groups. Given the consistency of our findings with Sannella’s analysis and the similarity of our findings to those in other awakening studies (Lockley, 2019; Woollacott et al., 2021), in Buddhist meditation (Lindahl et al., 2017), and in Christian prayer practices (St. Romain, 1991, 2017; Campbell, 1996; Poloma and Hoelter, 1998), it is possible that kundalini-related influences are broadly present in contemplative practices. A study of Buddhist practitioners (Lindahl et al., 2017) associated involuntary body movements with kundalini-like “surges of somatic energy.” Evangelical participants of Luhrmann and Morgain (2012) also reported phenomena like a “jolt of energy” or an “adrenaline surge” that remained unexplained in their study, and those phenomena were unrelated to the influence of imagination, repeated prayer practice and cultural support. Understanding what is involved in the expression of kundalini-related phenomena could offer an important new avenue through which to interpret spontaneous meditation and contemplative experiences. Thus, it will be important to identify objective correlates of kundalini-related activity.

While physiological mechanisms unrelated to the kundalini phenomenon may explain some of the experiences reported to us, the large prevalence of such experiences, including simultaneous experiences in multiple modalities, suggests the presence of a unique kundalini-related phenomenon. Many participants also reported spontaneous experiences with anomalous qualities, which are difficult to reconcile with models of kundalini that are associated with purely sensory qualities. Instead, traditional literature formulates kundalini as a broader phenomenon encompassing a large variety of spiritual experiences and nondual meditation states (e.g., Katyal, preprint).^1^ To derive a broader understanding of kundalini, and to potentially quantify the “depth” of kundalini experiences, future studies would need to be designed to investigate kundalini experiences using a broader range of questionnaires along with dimensional reduction approaches such as the Rasch scaling approach (Lange and Thalbourne, 2007; Lange, 2017; Padgett and Morgan, 2020), or other approaches used in modern Item Response Theory (Reise and Henson, 2003; Willoughby et al., 2011; Sauer et al., 2013; Choi and Asilkalkan, 2019).

Finally, we will briefly comment about two theories of how kundalini may be physically expressed. According to one theory (Bentov, 1988), kundalini-related experiences arise from brain structures resonating with breathing and heart rhythms. Bentov considered the traditional concept of rising spinal activity to be an illusion and considered the pattern of kundalini activity to follow the somatic representation of the primary somatosensory and motor cortex. Accordingly, he proposed that after rising from the feet and legs, somatic experiences would move upward through the spine and back, and from the head then progress to the face, throat and the abdomen. No one within our participants described a sensory progression moving from the face to the throat and then the abdomen. Two of the 15 participants in the KRSM group reported movement that was both up and down. However, both solely referenced the spine as the location of this process. Thus, our reports of rising sensations fail to follow Bentov’s suggested somatic representation.

Recently, one of us proposed an alternative mechanism for kundalini expression (Maxwell, 2009). According to it, neural and glial gap junctions within the spine and brain form compartments that can be linked. A joined sequence of these compartments could create the “channel” (*sushumna nadi*) that yogis have long described. Kundalini would be expressed as a state change moving through portions of this channel that have been successfully linked and opened. Gap junctions, also known as electrical synapses, have been shown to form functional compartments that pass electrical signals throughout the central nervous system (Rela and Szczupak, 2004), form syncytial groupings among glia (Nagy and Rash, 2000; Tonkin et al., 2015), enhance synchronous oscillatory firing in neurons (Maex and de Schutter, 2007; Hu et al., 2020) and contribute to the control of biosynthesis and release of secretory products from all glands (Meda, 2018), among many additional functions. While brain mechanisms certainly have relevance for kundalini-related experiences, if traditional Tantric Yoga perspectives are accurate, spinal and other peripheral features should be included in any theory of how kundalini is expressed.

### Limitations

One limitation of the present study is that the questionnaire was long, requiring about an hour to complete, which may have promoted brief responses and loss of detail. An alternative approach would have been to use phenomenological interviews to allow extended exploration of participants’ experiences. Another major limitation is that, since many of the experiences may have occurred for the participants at an unspecified time in the past, they would be vulnerable to misattributions, faulty memory and culturally supported expectations.

The subjective responses had much variability. Grammatical variants and strong synonyms were considered to be the same. However, due to potentially different connotations, many similar terms were tallied separately, diminishing some scores.

Even though the participants performed a uniform set of meditative practices, which is novel for kundalini-related research, the differences in durations for the various meditative practices performed by the participants may have added uncontrolled variability within the meditation variables.

The questions for the different modalities were not structured identically. They were designed to optimize understanding each individual modality. Separating the experiences by modality artificially isolated components of the full experience. Having greater comparability would foster expanded statistical analysis (such as through Rasch scaling, other Item Response Theory approaches and Structural Equation Modeling) which is recommended for future research on kundalini-related experiences.

Our findings are further limited by lacking systematic analysis of Sannella’s interpretive (i.e., cognitive distortions, major alterations of self-perspectives, and unusual or extreme affect) and non-physiological (i.e., anomalous and paranormal) experiences. This would be remedied by adding broader measures of positive and negative affect, as well as formal measures of self-awareness and anomalous experiences in future research.

## Conclusion

The spontaneous meditation experiences reported in this exploratory pilot study provide the first quantification in a community sample of the incidence of sensory, motor and mood characteristics of the kundalini signs and symptoms identified by Sannella (1992), and particularly a sample that has specifically trained on the same kundalini awakening practices. While we do not have a specific model to represent the form of kundalini’s experiential expression, the experiences that were reported to us resembled much of what Sannella suggested were associated with kundalini. The richness of the descriptive data that we collected will foster subsequent kundalini exploration. We observed that kundalini-related experiences in Tantric Yoga meditation were associated with mostly positive mood shifts, unlike past kundalini research, which until recently has been studied mostly from an adverse psychological perspective. We were unable to identify any significant relationship between our experiential modalities and the three meditation quantity variables. This indicates kundalini-related expression may involve inter-individual differences not mediated by the amount of meditation. We did observe, consistent with prior research, that greater meditation was associated with greater positive affect, less negative affect, and greater mindfulness. We also observed that participants having greater daily meditation (MDM) had significantly stronger scores for a set of supplementary yogic practices, representing adoption of a broader Tantric Yoga lifestyle.

## Data Availability Statement

The datasets presented in this study can be found in online repositories. The names of the repository/repositories and accession number(s) can be found at: Open Science Framework: https://osf.io/cnghp/.

## Ethics Statement

Ethical review and approval was not required for the study on human participants in accordance with the local legislation and institutional requirements. The participants provided their written informed consent to participate in this study in accordance with the standards of the American Psychological Association.

## Author Contributions

RM is the first author and conducted the research, analysis, and the majority of the writing. SK is the senior author and provided critical comments, edits, and context within the literature. All authors contributed to the article and approved the submitted version.

## Conflict of Interest

The authors declare that the research was conducted in the absence of any commercial or financial relationships that could be construed as a potential conflict of interest.

## Publisher’s Note

All claims expressed in this article are solely those of the authors and do not necessarily represent those of their affiliated organizations, or those of the publisher, the editors and the reviewers. Any product that may be evaluated in this article, or claim that may be made by its manufacturer, is not guaranteed or endorsed by the publisher.

## Footnotes

^1^ https://mindrxiv.org/ewb7z
