# Supplementary material for: Characteristics of Kundalini-Related Sensory, Motor, and Affective Experiences During Tantric Yoga Meditation
Source: Front Psychol. 2022 Jun 30;13:863091. doi: 10.3389/fpsyg.2022.863091 (PMC9282169; doi:10.3389/fpsyg.2022.863091)
Supplement: Supplementary file 1 [file Table_1.docx]

**Additional Methods Information**

**Additional Details Concerning Supplementary Practices**

Within Ananda Marga, yoga asanas are recommended to be performed twice daily. Participants specified the daily amount of time and the number of days per week asanas were performed. We considered asanas performed at least daily for any amount of time to be positive observance.

Ananda Marga practitioners are encouraged to follow strict dietary guidelines. This diet is known as a “sattvic” or “sentient” diet, meaning lacto-vegetarian or vegan, with additional exclusions, such as no onions, garlic and mushrooms. Participants indicated if they were following the sattvic diet, or not.

Ananda Marga practitioners are also advised to observe fasting (no consumption of food or water unless medically required) at regular intervals. This fasting procedure is observed for a 24-hour period on a day determined by the lunar cycle (11 days after every new moon and full moon; referred to as *Ekadashi*). Participants indicated if they were observing fasting on these occasions, or not.

In Ananda Marga, limiting sexual activity is considered to benefit meditative practice. Therefore, we asked the participants about the frequency of their sexual activity. Those who reported a monthly frequency or less were considered to meet a “moderation” standard and have a positive observance.

Drug use is discouraged in Ananda Marga. We asked participants to specify all recreational drug use, including alcohol and tobacco products, but excluding caffeine. Positive observance represented no use of any substance, other than caffeine.

**Data Analysis**

One way we attempted to clarify the uniqueness of our participant sample in comparison to other reports of kundalini-related experiences was by gathering descriptive data and calculating the incidence of characteristics specific to each modality. In addition to the descriptive data, we statistically compared the incidence of each modality with the remaining modalities. We did this to identify relationships that might foster further predictions about mechanisms generating the modality experiences. We utilized six logistic regressions for this purpose, each modality being used once as the dependent variable. We corrected significance levels for each logistic regression using the “FDR” (False Discovery Rate) procedure (Benjamini and Hochberg, 1995). We used similar logistic regressions to compare each modality with measures of the quantity of meditation, Supplementary Practices. We also used similar logistic regressions to compare each modality with measures of trait mindfulness, trait positive and negative affect. We used logistic regressions to determine whether invariance was present for gender and linear regressions for age, and considered them adequate given our largely descriptive exploratory data.

To determine the accuracy of our prediction that increasing quantity of meditation would be associated with increased trait mindfulness and trait positive affect, we used partial correlations in which the effect of age was removed. Age is known to influence affect and happiness (Carstensen et al., 2011; Yang, 2008). The levels of significance for these partial correlations were also FDR-adjusted.

Data used for multivariable statistical analysis had 2.33% of responses missing. None of the essential demographic and meditation variables had any missing values. Descriptive responses associated with the modalities had additional missing data which was not included in the missing data tally. All missing responses used for multivariable statistical analysis were imputed to facilitate statistical comparison of variables. There is greater accuracy and less bias in data that has been multiply imputed compared to data using the common approaches of pairwise or listwise deletions (Newman, 2014).

We conducted statistical analysis of the data using R (v.3.6.1) statistical software and RStudio (v. 1.2.5001). For imputation, we used the “mice” (Multivariate Imputation and Chained Equations) package. For logistic and linear regressions, we used the R native “stats” package. For the partial correlations and test reliability, we used the “psych” package. The selection of variables used for statistical analysis in R (Survey 1 data for R.csv), and the data file used for the imputation (Survey 1 for Impute.csv) may be found at <https://osf.io/cnghp/>.

**References**

Carstensen, L. L., Turan, B., Scheibe, S., Ram, N., Ersner-Hershfield, H., Samanez-Larkin, G. R., et al. (2011). Emotional Experience Improves With Age: Evidence Based on Over 10 Years of Experience Sampling. *Psychology and Aging* 26, 21–33. doi: 10.1037/a0021285.

Newman, D. A. (2014). Missing Data: Five Practical Guidelines. *Organizational Research Methods* 17, 372–411. doi: 10.1177/1094428114548590.

Yang, Y. (2008). Social Inequalities in Happiness in the United States, 1972 to 2004: An Age-Period-Cohort Analysis. *American Sociological Review* 73, 204–226.
